# Pollinator specialization increases with a decrease in a mass‐flowering plant in networks inferred from DNA metabarcoding

**DOI:** 10.1002/ece3.5531

**Published:** 2019-09-30

**Authors:** André Pornon, Sandra Baksay, Nathalie Escaravage, Monique Burrus, Christophe Andalo

**Affiliations:** ^1^ Laboratoire Evolution et Diversité Biologique UMR 5174 CNRS IRD Université Toulouse III Paul Sabatier Toulouse France

**Keywords:** DNA metabarcoding, food niche breadth, individual pollination network, mass‐flowering species, pollinator generalization, pollinator specialization, species pollination networks

## Abstract

1. How native mass‐flowering plants affect the specialization of insects at individual and species levels and the consequences for pollination networks have received much less attention than for mass‐flowering crops or alien species and basically remain unexplored.
2. Using existing DNA metabarcoding data on the pollen loads of 402 flower‐visiting insects, we assessed the effects of a native mass‐flowering plant of high reward quality, the shrub *Rhododendron ferrugineum*, on pollination networks by investigating: (a) the food niches of individual pollinators and pollinator species and (b) the structure of individual and species networks in subalpine heathland patches with extremely contrasted densities of *R. ferrugineum*.
3. Relative to its high abundance in high‐density patches, the shrub was greatly underrepresented and did not dominate individual's or species' generalized networks, rather individual and species specialization increased with a decrease in *R. ferrugineum* density. Furthermore, individuals of the more generalist dipteran *Empididae* species tended to extend exclusive interactions with rare plant species in low‐density networks. The same trend was observed in the more specialist *Apidea* but toward rare species in high‐density networks. Our results reveal a quite paradoxical view of pollination and a functional complementarity within networks. Niche and network indices mostly based on the occurrence of links showed that individual pollinators and pollinator species and networks were highly generalized, whereas indices of link strength revealed that species and above all individuals behave as quite strict specialists.
4. *Synthesis*. Our study provides insights into the status of a native mass‐flowering plant in individual's and insect species' food niches and pollination networks. It revealed that a generalist pollinator species can be highly specialized at the individual level and how rare plant species coexisting with mass‐flowering plants may nevertheless be visited.

## Graphical abstract

Using metabarcoding and thus the strength of plant–pollinator links, we provide new strong insights into the status of a native mass‐flowering species (MFS; compared to crops or aliens species) in individual and species pollination networks and the general specialization of generalist individuals increasing with the decrease in the MFS abundance.

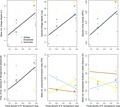

## 1 INTRODUCTION

Many plant communities contain crop, alien, or native mass‐flowering plants usually distributed in densely populated patches alternating with areas where they are rare or absent. Studies of agroecosystems and of invaded areas have shown that crop and invasive mass‐flowering plants can (a) attract pollinators and monopolize visits (Holzschuh et al., 2016; Kovács‐Hostyánszki et al., 2013; Meyer, Gaebele, & Steffan‐Dewenter, 2007) from the surrounding area; (b) be dominant or important in shaping pollination networks (Lopezaraiza‐Mikel, Hayes, Whalley, & Memmott, 2007; Magrach, González‐Varo, Boiffier, Vilà, & Bartomeus, 2017; Stanley & Stout, 2014; Tiedeken & Stout, 2015) particularly under high levels of invasion (Kaiser‐Bunbury, Valentin, Mougal, Matatiken, & Ghazoul, 2011); and (c) often (but not always) have negative effects on coflowering native species (Jakobsson, Padron, & Traveset, 2008; Magrach et al., 2017). Yet, how the native mass‐flowering plants affect the specialization of insects at individual and species levels and what the consequences for pollination networks have received much less attention and basically remain unexplored.

Since pollinators tend to preferentially visit the most abundant and/or rewarding resources in a given area (Dauber et al., 2010; Ghazoul, 2005), native mass‐flowering plants could, like exotic plants, attract most pollinators from the surroundings and, to a certain extent, dominate the networks with possible negative effects on coflowering species. However, because pollinator behavior may change with the abundance and the quality of resources even in small areas (Dupont et al., 2014; Osborne & Williams, 2001), the reduction in mass‐flowering plant cover in the community could lead to a new pollination network structure, particularly if the reduction results in a dramatic decrease in locally available resources. On the one hand, lower floral resource abundance may favor generalist species that can compensate for the decrease in mass‐flowering species by using alternative resources and/or keep species specialized on the mass‐flowering plants away from the vegetation patch. What is more, individuals may broaden their diet (individual generalization) in response to competition for food (Fontaine, Collin, & Dajoz, 2008; Kunin & Iwasa, 1996). Together, these processes would lead individual and species networks to be more generalized with high connectance (the proportion of links that actually occur in networks, which increases with generalization) and possibly high nestedness (the degree to which specialist plants and insects interact with generalists plants and insects). On the other hand, in response to competition for resources, a subset of individuals may shift to other resources (individual specialization) even though other members of the population continue to forage on the original resources (Bolnick et al., 2003; Svanbäck & Bolnick, 2007). Individual specialization on different floral resources would lead to generalized species networks.

Whether species networks mainly comprise either specialist or generalist individuals would have very different ecological consequences. Indeed, besides affecting the population dynamics of the pollinators themselves (Araújo, Bolnick, & Layman, 2011), the magnitude of individual specialization may strongly determine the quantity and the quality (conspecific vs. heterospecific) of pollen transferred and have considerable consequences for both plant fitness and population and community dynamics. Furthermore, generalized species networks with high connectance and nestedness are considered to be less vulnerable to species losses (Memmott, Waser, & Price, 2004). Thus, understanding the network architecture and whether the species mainly comprises specialized or generalized individuals is essential since it can affect the structure and the robustness of the network, community stability, and pollination services in the face of disturbances (Thébault & Fontaine, 2010).

In this study, we focused on the effects of the high reward native mass‐flowering shrub *Rhododendron ferrugineum* on the structure of pollination networks by investigating: (a) individual pollinator's (a total of 402) and pollinator species' food niches and (b) the structure of individual and species networks in subalpine heathland patches with highly contrasted floral densities of *R. ferrugineum*. We investigated pollinator food niches and networks by using DNA metabarcoding data that were obtained in a previous study at the site (Pornon, Andalo, Burrus, & Escaravage, 2017). Metabarcoding has already been shown in several studies to successfully identify plant taxa in pollen mixtures (Cornman, Otto, Iwanowicz, & Pettis, 2015; Galimberti et al., 2014; Galliot et al., 2017; Lucas et al., 2018; Macgregor et al., 2019; Pornon et al., 2016; Richardson et al., 2019) at a higher taxonomic resolution than microscopy (Vamosi, Gong, Adamowicz, & Packer, 2017). A recent meta‐analysis (Lamb et al., 2018) concluded on a significant quantitative positive linear relationship between the proportion of sequence reads and the proportion of species in original mixtures from very different origins (stomach contents, feces, airborne pollen, etc.). Some studies provided evidence for the quantitative potential of pollen metabarcoding both with nuclear (ITS) and plastid markers (rbcL, trnL, trnH; Keller et al., 2015; Kraaijeveld et al., 2015; Pornon et al., 2016; Richardson et al., 2019; Smart et al., 2017). However, Bell et al. (2018) and Richardson et al. (2019) obtained less reliable results with ITS2. Furthermore, depending on the species, higher or lower proportions of sequence reads may be found than expected (Bell et al., 2018). Despite these limitations, in a simulation study, Deagle et al. (2018) showed that even with methodological biases are included, sequence counts can provide a more reliable description of species diet in many scenarios than link occurrence alone. Moreover, since pollen grains may accumulate on insect bodies during successive visits (Delmas, Fort, Escaravage, & Pornon, 2016; Jacobs et al., 2010), the proportion of either pollen grains or sequences of a given plant species in an insect pollen load may reflect the proportion of visits to the species (Pornon et al., 2016; Stanley & Stout, 2014).

In heathland patches with a high density of *R. ferrugineum* and limited alternative resources, we predicted (H1) that individual insects would forage mostly on the shrub. Thus, individuals, species, and networks would mainly be specialized in *R. ferrugineum* with low between‐individual diversity in pollen use, large food niche overlap between individuals or species, and that the individual's diet would largely resemble the species' diet. However, this overall tendency could, to some extent, be counteracted if *R. ferrugineum* is not their favorite source of food, if it provides resources that are difficult for some insect species to access, or if individual and/or colony development require diversified resources (Girard, Chagnon, & Fournier, 2012; Tasei & Aupinel, 2008). In patches with low *R. ferrugineum* density and limited alternative resources, pollinators could compete for less available flower resources (Table 1). Delmas et al. (2016) observed more frequent visits to *R. ferrugineum* and smaller quantities and proportions of its pollen in insect loads in low‐density patches than in high‐density patches, suggesting that pollinators had to share fewer resources among more individuals in low‐density patches. We hypothesized that in response to global resource depletion, individuals would either diversify their diet (H2: higher individual generalization) or specialize in different floral resources (H3: higher individual specialization) with different consequences for individual and species networks.

**Table 1:** Characteristics of vegetation in heathland patches

	Heathland patches			
	LDP1	LDP2	HDP1	HDP2
**Patch area (ha)**	0.17	0.6	2	15.8
**Community structure and diversity**				
*Rhododendron ferrugineum* cover (%)	0.93	3.2	98	69.5
Surrounding community cover (%)	99.07	96.8	2	30.5
Number of species in SC	32	11	12	16
**Floral density (per m^2^)**				
*R. ferrugineum*	27	107	2,788	2,273
Surrounding community	273	41	0.84	7
Total	300	148	2,789	2,280
**Cover of dominant species (%)**				
*Anthyllis vulneraria*	4	0	0	0
*Cardamine pratensis*	2.6	15.7	0	0.1
*Chamaespartium sagittale*	7.2	0	0	0
*Dactylorhiza sambucina*	2.3	0	0	0
*Genista pilosa*	2.3	4.2	0	0
*Hippocrepis comosa*	3.9	0	0	0
*Lotus corniculatus*	2	3.7	0	0.1
*Polygala calcarea*	47.5	0	0	0
*Potentilla erecta*	1.5	1.4	0	0
*Ranunculus acris*	1.7	1.3	0	0
*Trifolium endressi*	12.4	0	0	0

Note

Only species with a cover of more than 1% in a given patch are listed.

Abbreviations: HDP1 and HDP1, patches with high floral density; LDP1 and LDP2, patches with low floral density; SC, the surrounding community.

## 2 MATERIALS AND METHODS

### 2.1 Study site and data collection

We sampled plants and their visiting insects in four subalpine *R. ferrugineum* heathland patches within a 3‐km^2^ area in the French Central Pyrenees (southern France) near the village of Camurac (42°46′31″N 01°55′45″E; 1,660 m a.s.l.) during the shrub's blooming period (June 2012). Heathlands were spatially delimited in visually distinct patches (i.e., aggregations of *R. ferrugineum* embedded in their associated flowering community; hereafter referred as “surrounding communities”) and separated from other patches by a meadow. *R. ferrugineum* is an 0.7–0.8‐m high evergreen shrub that grows as isolated individuals in vast subalpine meadows and in many other locations, covering hundreds of hectares (Pornon, Escaravage, Till‐Bottraud, & Doche, 1997). It bears up to 3,000 red, weakly zygomorphic tubular flowers per m^2^ (Pornon et al., 1997) that produce nectar (0.7–1.4 μl; Escaravage, Pornon, Doche, & Till‐Bottraud, 1997) with a high sugar concentration (mean 63.4° Brix in our study site; Delmas, Escaravage, & Pornon, 2014). It is visited by many pollinators among which hymenoptera are the most typical (Delmas et al., 2014). The main characteristics of the vegetation in the patches are listed in Table 2. The four patches (low‐density patches, LDP1 and LDP2; high‐density patches, HDP1 and HDP2) had different floral densities (and different floral resources), mostly determined by *R. ferrugineum* cover. The distance between the patches LDP1 and LDP2 was 800 m, 1,500 m between the patches HDP1 and HDP2 and varied between 1,250 and 1,750 m between LDP and HDP. Since the foraging distance of most pollinators (including large bumblebees) is usually shorter than 500 m (Gathmann & Tscharntke, 2002; Zurbuchen et al., 2010), data obtained in the four patches can be considered independent.

**Table 2:** Network and food niche indices and their ecological significance

	Ecological significance
**Network‐level indices**	
Connectance (C)	The proportion of possible links actually realized in a network. C decreases with specialization and network size
Nestedness	Specialization asymmetry: the degree to which specialists (plants and insects) interact with generalists (plants and insects). High nestedness is generally considered to increase network robustness in the face of perturbations
Interaction evenness (E)	Evenness of interaction frequencies between different pairs of species. The lower the evenness, the higher the specialization in the network
$H_{2}^{\prime }$	Degree of network specialization
Kullback‐Leibler distance *d*′	Degree of individual or species specialization in networks. The level of exclusiveness of an insect species (in species networks) or an individual (in individual networks) on rare plant species
**Food niche indices**	
Mean linkage degree of individual (*L_i_*) or species (*L*_sp_) pollinators	Mean diet breath of individual or species pollinators, respectively
Species *L*_sp (80%)_ or individual *L_i_*_ (80%)_	The degree to which species or individuals specialize in some species among all the plant species in their food niche
Within (WIC)‐ versus between‐individual component (BIC) of population food niche	Proportion of the total food niche width (TNW) of the population explained by intraindividual versus interindividual diet diversification
Interindividual diet overlap (IO)	The extent to which individual insects share and potentially compete for the same plant resources
Interspecies diet overlap (SPO)	The extent to which species share and potentially compete for the same plant resources
Proportional niche similarity (PS*_i_*)	The extent to which conspecific individuals use the same resources as their population

*Rhododendron ferrugineum* floral density (mean no. flowers per m^2^) was estimated from the shrub cover and measured in a 400 m^2^ plot randomly chosen in the center of the patch, and the number of flowers was counted in 0.25 × 0.25 m plots placed on 20 randomly chosen individuals (Delmas et al., 2014). In each patch, the floral density of each species in the surrounding community was estimated three times during the *R. ferrugineum's* blooming period (early, mean, and late) in twenty 50 × 50 cm plots haphazardly chosen in the patches. Floral density was either the number of flowers or, for species bearing countless tiny flowers such as *Asteraceae*, *Alchemilla* sp. the number of inflorescences per m^2^.

In each patch, pollinators were captured twice a day (from 10:00 to 11:30 a.m. and from 2:00 to 3:30 p.m.) on two consecutive sunny days (i.e., a total of 6 hr per patch) in a 625 m^2^ area (25 m × 25 m) located in the center of each patch. This methodological decision was taken based on previous data obtained in the same site by Delmas et al. (2014). Insects were captured along haphazard walks in the patch. Only insects in contact with fertile parts of the flower were captured, and clean new nets were used for each capture. Insects were placed in clean scintillation tubes and stored at 4°C in the field, then at −20°C in the laboratory. The species of plant on which each insect was captured while foraging was recorded. Overall, 402 insects were captured and the pollen they carried was identified using DNA metabarcoding and markers adapted to possibly degraded DNA in pollenkitt, namely the chloroplastic marker P6 loop of *trnL* (UAA) intron and the nuclear *ITS1* marker. Pollen removal from insect bodies, DNA metabarcoding procedures, field and laboratory practices applied to prevent sample contamination, sequencing errors, and potential misidentifications are detailed in Pornon et al. (2017) and Pornon et al. (2016).

### 2.2 Individual and species pollination matrices

For each patch, we completed individual pollinator–plant species matrices (*i*‐sp M = [*n_ij_*]_I × P_; I: insects, P: plants) with *n_ij_* in each cell being the largest number of either *trnL* or *ITS1* sequences (*i*‐sp M_seq_) or the presence/absence (1; 0 otherwise) of sequences (*i*‐sp M_link_) of the plant species *j* yielded from the pollen load of the individual insect *i*.

Thus, *i*‐sp M_link_ recorded only the presence–absence of individual pollinator–plant links, whereas for each link, *i*‐sp M_seq_ recorded the number of sequences as a surrogate for the number of pollen grains carried by the pollinator and thus providing a rough estimate of the strength of the link. The rationale behind the choice of considering the largest number of either *trnL* or *ITS1* sequences was to keep in matrices the results of the marker whose amplification was the most successful, in aim to prevent the underrepresentation of some species. Indeed, it is often observed during PCR that a species may be properly amplified by one marker but not by another one (Pornon et al., 2016). For both *i*‐sp M_seq_ and *i*‐sp M_link_, we took a count of more than 1,000 sequences (i.e., *n_ij_* > 1,000 seq.) from a given plant species as a proof of a link with the aim of removing, as far as possible, contaminations that could for instance have occurred due to airborne pollen or nonpollen plant tissues deposited on insect bodies. Therefore, all values lower than or equal to 1,000 sequences were set to zero in *i*‐sp M_seq_ and *i*‐sp M_link_, and the networks. The rationale behind the choice of a 1,000 sequence threshold was that, since insects move, rest and nest (e.g., *Bombus* sp.) in the grassland matrices and as wind‐pollinated plants usually produce far more abundant airborne pollen than insect‐pollinated plants, grasses should represent a level of widespread background contamination that insect‐pollinated plants would barely, if ever, reach. In a previous study (Pornon et al., 2017), we observed that the 1,000 sequence threshold allowed the removal of more than 95% of grass sequences, and under all hypotheses, also removed all potential contamination originating from insect‐pollinated plants. From *i*‐sp M_seq_ and *i*‐sp M_link_, we analyzed the following: (a) individual and species pollination networks and (b) individual and species food niches.

### 2.3 Network‐level analysis

For each patch, we built (a) individual pollinator–plant species networks (*i*‐sp N) by extracting submatrices (one per insect species for every species represented by at least two individuals) from *i*‐sp M_link_ (*i*‐sp N_link_) or *i*‐sp M_seq_ (*i*‐sp N_seq_); (b) bipartite pollinator species–plant species networks (sp‐sp N) by summing either the links (sp‐sp N_link_) in *i*‐sp M_link_ or the number of sequences (sp‐sp N_seq_) in *i*‐sp M_seq_ of all individuals of the same insect species. Using the R “bipartite” package (R Core Team, 2013; version 1.17; Dormann, Fründ, Blüthgen, & Gruber, 2009), for the sp‐sp N and *i*‐sp N of each patch, we calculated (a) connectance, (b) nestedness (100−*T*)/100, with *T* = temperature; ranging from 0 (chaos) to 1 (perfect nestedness), (c) interaction evenness (*E*; based on Shannon's index which is a measure of the skewness in the distribution of interaction frequencies), (d) the degree of network specialization ($H_{2}^{\prime }$), and (e) the degree of species (sp‐sp N) or individual (*i*‐sp N) pollinator specialization through the standardized Kullback‐Leibler distance *d*′ (Vázquez, Blüthgen, Cagnolo, & Chacoff, 2009) and *d'* for plant species. $H_{2}^{\prime }$, *d*′ and *E* range from 0 to 1 a value of 1 indicating perfect specialization ($H_{2}^{\prime }$, *d'*) and perfect frequency evenness (*E*). The ecological relevance of these parameters is explained in Table 2, and their underlying statistics and mathematics are detailed in Appendix S1 and in Blüthgen, Fründ, Vázquez, and Menzel, (2008). In order to determine whether the network structure differed beyond what would be expected due to random effects, 1,000 null networks (Dormann et al., 2009) were generated using Patefield's algorithm implemented in the nullmodel function or, for *i*‐sp N_link_ specifically, the *mgen* function (with the options rep.cell set to false and autotransform set to equiprobable) of the bipartite package (Dormann, Gruber, & Fründ, 2008). Null networks were constructed through random sampling of our empirical matrices constraining the marginal totals. Thus, the relative frequencies of species in empirical matrices were conserved in null matrices. However, with the *mgen* function interaction assignment in the null network still depended on marginal probabilities but the binary nature of the original *i*‐sp N_link_ matrices was kept. All the above‐mentioned indices were assessed for the 1,000 null networks, and the mean and 95% confidence intervals were calculated.

### 2.4 Food niche analysis

We calculated the mean linkage degree of individual (*L_i_*) and species (*L*_sp_) pollinators, that is, the mean number of plant species with which an individual insect or a species interacts. To assess whether and to what extent individual or species pollinators preferred some plant species over others, we calculated the minimum number of plant species (and therefore links) needed to account for 80% of the sequence reads for a given individual, hereafter noted *L_i _*_(80%)_ or species, hereafter *L*_sp (80%)_. The greater the difference between *L*_sp (80%)_ and *L*_sp_ and between *L_i_*_ (80%)_ and *L_i_*, the smaller the number of plant species preferred by species and individuals, respectively (higher specialization). The specific attractiveness of *R. ferrugineum* was evaluated through the proportion of individual insects carrying its sequences.

Following Bolnick, Yang, Fordyce, Davis, and Svanbäck (2002), we estimated the within‐individual component (WIC is the variation in pollen types used by an individual) and the between‐individual component (BIC: variation in pollen types used among individuals) of the total niche width (TNW) of the population (with TNW = WIC + BIC). The relative individual specialization (WIC/TNW) tends to 1 when each individual (generalist) uses the full range of pollen types used by the population and tends to 0 when each individual (specialist) uses a small subset of the population resources. We estimated individual and species niche segregation by calculating the interindividual (IO) and species (SPO) food niche overlap, that is, the proportion of overlap in pollen use either among conspecific individuals or among the species of the pollinator community. Finally, we calculated the proportional niche similarity between individuals and their populations (PS*_i_*). The indices ranged from 0 (total segregation) to 1 (total overlap). The ecological significance of these indices is summarized in Table 2, and their mathematical bases are detailed in Appendix S1. The proportion of individuals carrying *R. ferrugineum* pollen, *L_i_*, *L*_sp_, SPO, and IO, were obtained from binary data (M_link_), whereas *L*_sp (80%)_, *L_i _*_(80%)_, BIC, WIC, TNW, and PS*_i_* were based on sequence counts (M_seq_).

### 2.5 Effects of resource density on food niches and networks

We analyzed the effects of *R. ferrugineum* floral density, taxonomic insect families, and their interactions on network and on food niche indices using mixed linear models fitted with the HLfit function of the spaMM package (Rousset & Ferdy, 2014) and the prior.weights option that accounts for the different sizes of samples. We examined individual indices at family (*Apidae*, *Empididae*, and *Syrphidae*) instead of at species level since some species had either too few individuals or were present in only one patch. To control for spatial autocorrelations, a random patch effect was added to the models. Residuals normality was always checked, and Box–Cox (Box & Cox, 1964) power transformation was applied if required. For the proportion of individuals carrying *R. ferrugineum* sequences, a binomial error was applied.

## 3 RESULTS

### 3.1 Characteristics of the interacting communities

We captured, respectively, 97 and 123 insects in the low‐density LDP1 and LDP2 patches and, respectively, 82 and 100 in the high‐density HDP1 and HDP2 patches, belonging to, respectively, 29, 25, 25, and 29 species (Table S1) among which *Apis mellifera*, *Bombus lucorum*, *B. wurflenii*, *Empis leptempis pandellei*, *E. l. tessellata*, *Sphaerophoria batava*, and *Volucella bombylans* were the most abundant (53% of sampled insects). Thus, although there were almost 20 times fewer floral resources in the low‐density LDP2 patch than in the high‐density HDP1 and HDP2 patches (Table 1), more insects were captured in the low‐density LDP2 patch. We identified, respectively, 39 and 54 plant species in the insect pollen loads in the low‐density LDP1 and LDP2 patches and 61 plant species in high‐density HDP1 and HDP2 patches corresponding to 1.2, 4.9, 5.1, and 3.8 times the number of plant species surveyed in the 625 m^2^ plots. Therefore, pollinators foraged far beyond the area in which they were observed foraging.

*Rhododendron ferrugineum* accounted for only 4.6%, 10.8%, 9.6%, and 7.9% of insect links in sp‐sp N_link_ and 3%, 20.6%, 30%, and 15.5% in sp‐sp N_seq,_ in the low‐density LDP1 and LDP2 patches and in the high‐density HDP1 and HDP2 patches, respectively. However, 27.8% and 43.9% of insects were captured when foraging on the shrub in the low‐density LDP1 and LDP2 patches and 41.6% and 41.9% in the high‐density HDP1 and HDP2 patches, respectively. Thus, as previously demonstrated (Pornon et al., 2017) metabarcoding unveiled many links that existed both outside the study plots and before the capture, which therefore could not be observed during the visit surveys. Despite being visited by a broad array of insect species (sp‐sp N_link_), *R. ferrugineum* was abundant only in the pollen loads of *Apis mellifera*, *Bombus lucorum*, and *B. pratorum* (Figure S1). Most individuals of some species (*Volucella bombylans*, *Empis leptempis pandellei*, *Bombus wurflenii*; Figures S1 and S2) carried small quantities of *R. ferrugineum* pollen (as inferred from the DNA sequence number), whereas several species generally carried no pollen from the shrub (Figure S2), apart from rare individuals (*S. batava*, *S. scripta*, *E. e. tessellata*) carrying a minute quantity. In surrounding communities, social bees mainly visited plants with yellow zygomorphic flowers (*Cytisus scoparius*, *Lotus corniculatus*, *Genista pilosa*, *Hyppocrepis comosa*) and Diptera and solitary bees mainly visited plants with actinomorphic flowers (*Conopodium majus*, *Cardamine pratensis*, *Ranunculus polyanthemoides*, *Potentilla erecta*). *Geranium sylvaticum*, *Thalictrum aquilegiifolium*, *Bistorta officinalis*, and *Trollius europaeus* were only surveyed in high‐density patches and, accordingly, were only found in insect pollen loads in these patches. The total absence of *Polygala calcarea* in insect pollen loads of low‐density LDP1 is worth noting, even though this species accounted for half the total floral density.

### 3.2 Structure of species networks

Because of their large size, species networks (sp‐sp N_link_ and sp‐sp N_seq_) had low connectance (ranging from 0.12 to 0.17) and high nestedness (0.83–0.88). Species networks based on link occurrence (sp‐sp N_link_) were highly generalized (Table [Link], [Link]), both globally ($H_{2}^{\prime }$ ≤ 0.23) and at the species level (*d*′ insects and *d*′ plants generally ≤0.31) with relatively high interaction evenness (0.67–0.72). However, species networks based on sequence read counts (sp‐sp N_seq_) were 2.5 (low‐density LDP2 patch) to 3.5 (high‐density HDP2) times more specialized ($H_{2}^{\prime }$) than sp‐sp N_link_, as were insect species (*d'* insects: 1.7–2.3 times higher) and plant species (*d'* plants: 1.6–2 times higher) species. Consequently, interaction evenness was lower in sp‐sp N_seq_ (0.42–0.49) than in sp‐sp N_link_ (0.67–0.72; Table S2).

### 3.3 Structure of individual networks

Due to their far smaller size, individual networks (*i*‐sp N_link_, *i*‐sp N_seq_) had higher connectance (0.2–0.87) than species networks. *I*‐sp N_link_ were generalized (*d'* insects: 0–0.38; *d'* plants: 0–0.17; evenness: 0.68–0.93) and *i*‐sp N_seq_ comparatively more specialized ($H_{2}^{\prime }$: generally >0.5; *d'* insects: 0.015–0.70; *d'* plants: 0.007–0.56; evenness: 0.19–0.62).

Linear models showed that *i*‐sp N_seq_ of *Empididae* were more generalized (lower $H_{2}^{\prime }$; *p* = .0251) and individual in *Syrphidae*'s *i*‐sp N_seq_ more specialized (higher *d'*, *p* = .0063) than those of other families. Nestedness of *i*‐sp N_seq_ decreased as follows: *Syrphidae* > *Empididae *> *Apidae* (*p* = .0373).

### 3.4 Characteristics of individual and species food niches

**Figure 1:**
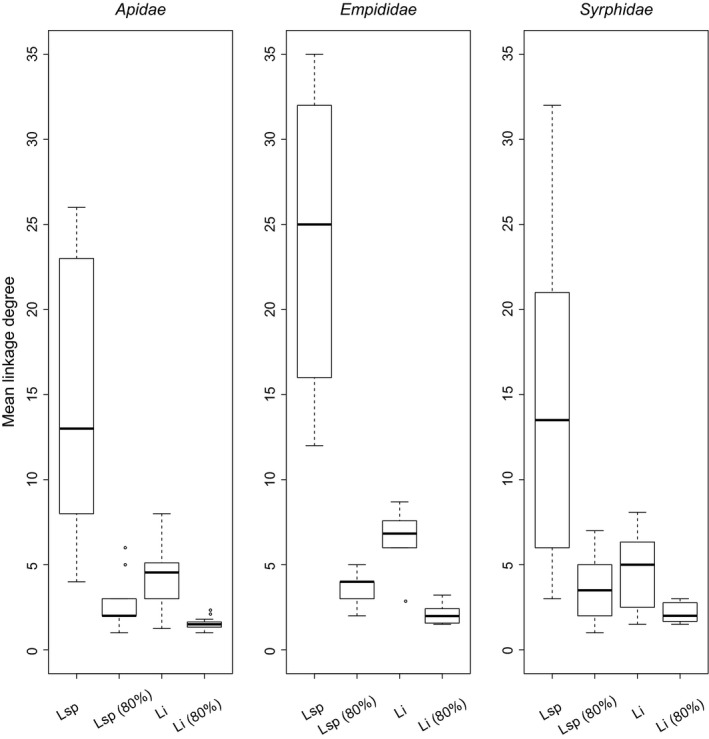
Change in linkage degrees from community to individual pollinators. *L*_sp_ and *L_i_*: species and individual linkage degrees; *L*_sp (80%)_ and *L_i _*_(80%)_: minimum number of links required to gather 80% of the pollen load of a pollinator species and of each individual, respectively. Box‐and‐whisker plots represent values for all combinations of patches × species

Pollinator species had large food niches, with mean species linkage degree (*L*_sp_) ranging from 3 to 35 (Table S2), but very low mean niche overlap (SPO = 14% ± 0.012). Furthermore, they had a highly uneven diet with preferential use of a small number of plant species (*L*_sp (80%)_: 1–7 species; Figure 1). Individual pollinators were also relatively generalized (*L_i_*: 1.25–7.6), although to a lesser extent than the species. They had very few preferred plant species (*L_i _*_(80%)_:1–3.2) and very low diet overlap (IO = 13% ± 0.025). On average, within‐individual variation in pollen type use accounted for 53% of the total species niches (WIC/TNW = 0.53 ± 0.031 SE; TNW = 1.3 ± 0.10; WIC = 0.68 ± 0.058). The proportional niche similarity between individuals and their populations was relatively low (PS*_i_* = 0.49 ± 0.016). In summary, these results revealed that, despite visiting a relatively broad range of plant species and thus having large diet breadth (and can thus be considered as generalists), each species, or even more strikingly each individual, was strongly specialized on a few and specific floral resources, most of which differed from those used by other species or conspecifics.

**Figure 2:**
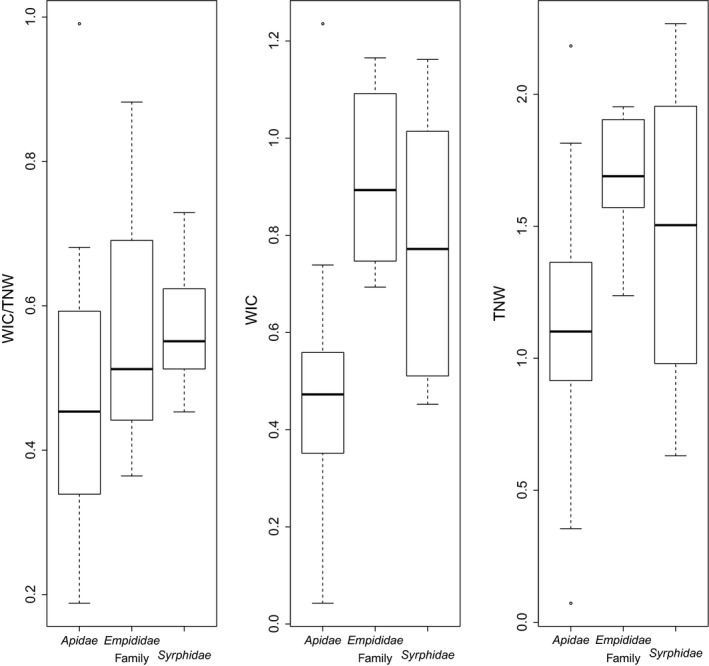
Variation in food niche breadth among pollinator families. SPO, species food niche overlap; TNW, total niche width; WIC, within‐individual component of niche breadth

Within this general trend, linear models highlighted differences between taxa with respect to (a) individual (*L_i_*, *p* = .0003; Figure 1) and species (*L*_sp_, *p* < .0038) diet breath, (b) the number of plant species preferentially foraged by individuals (*L_i _*_(80%)_: *p* < .0001) or species (*L*_sp (80%)_ *p* = .0142), (c) the indices of within‐individual variation in pollen type use (WIC; *p* = .0067), of total niche width (TNW, *p* = .0027; Figure 2) and of the WIC/TNW ratio (*p* = .0345), and (d) species niche overlap (SPO; *p* = .0086; Figure 2). *Empididae* and *Apidae* were the two taxa mostly responsible for these differences, the former being the most generalist and the latter the most specialist of the pollinator assemblage often at both individual and species levels. Thus, relative to other taxa, *Empididae* had higher *L_i_*, *L*_sp_, TNW, WIC, and SPO. In contrast, *Apidae* had lower *L*_sp (80%)_, *L_i _*_(80%)_, TNW, WIC, and WIC/TNW ratio than the other taxa.

### 3.5 Impact of the mass‐flowering plant on food niches and networks

**Figure 3:**
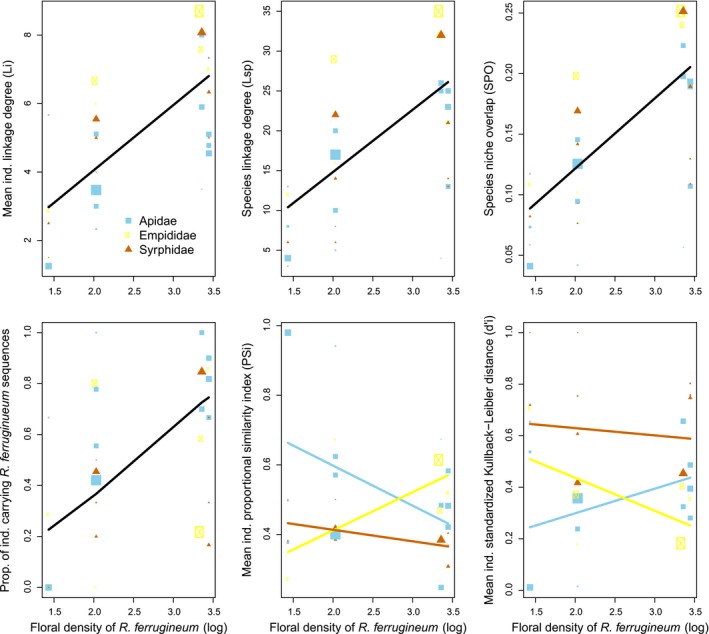
Effects (mixed linear model) of *Rhododendron ferrugineum* floral density on species and individual food niches and networks. Black lines show the independent effects of floral density, and the colored lines show family‐floral density interaction effects. The size of the symbols is proportional to the size of the sample

We observed overall higher specialization of pollinators in low‐density patches than in high‐density patches. Indeed, individual (*L_i_*, *p* = .0086) and species (*L*_sp_, *p* = .0186; Figure 3) diet breath, the number of plant species with which individual insects preferentially interacted (*L_i _*_(80%)_; *p* = .0356; Figure 3), and species niche overlap (SPO, *p* = .0082) tended to decrease with decreasing *R. ferrugineum* density. The same tendency was observed for the proportion of insects carrying *R. ferrugineum* pollen (*p* = .0145).

However, we also detected taxa‐floral density interactions, that is, higher niche individual‐population niche similarity (PS*_i_*) in low‐density patches for *Apidae*, whereas the opposite was observed for *Empididae* (interaction between patch density and insect family, *p* = .0198; Figure 3). Consistently, the *d'* of *Apidae* individuals tended to decrease and the *d'* of *Empididae* individuals tended to increase in low‐density networks compared to those in high‐density networks (interaction between patch density and insect family, *p* = .0473; Figure 3). Therefore, *Empididae* individuals tended to extend exclusive interactions with rare plant species in low‐density networks, whereas individuals *Apidae* did the same in high‐density networks.

## 4 DISCUSSION

In this study, we investigated the impact of the native mass‐flowering *R. ferrugineum* on individual and species networks and on the food niches of the most abundant pollinators by identifying and quantifying insect pollen loads by metabarcoding. We predicted that individual insects and insect species as a whole would mainly forage on *R. ferrugineum* in high‐density patches (Dauber et al., 2010; Ghazoul, 2005; H1) and would be either more generalized (Fontaine et al., 2008; Kunin & Iwasa, 1996; H2) or more specialized (Bolnick et al., 2003; Svanbäck & Bolnick, 2007; H3) in response to competition for limited resources in low‐density patches. Our results failed to confirm either H1 or H2, whereas, in agreement with H3, pollinators were clearly more specialized in low‐density patches than in high‐density ones.

### 4.1 The absence of insect specialization on the mass‐flowering plant

There are several possible nonexclusive explanations for the absence of pollinator specialization or constancy on *R. ferrugineum* in high‐density patches: First, based on the proportion of insects captured while visiting *R. ferrugineum* (41.8% in HDP compared to 8.5% of links inferred from metabarcoding), one could have concluded that pollinator specialization was greater. Thus, specialization was lower than expected under H1 simply because metabarcoding revealed many links that occurred both outside the study plots and before our sampling period (Pornon et al., 2017). Second, pollinators, in particular colonial species, require diverse resources and often high‐quality pollen (Somme et al., 2015) for individual and/or population development (Girard et al., 2012; Tasei & Aupinel, 2008). The possible poor quality of *R. ferrugineum* pollen or nectar as demonstrated in other ericaceous species (low polypeptide and total amino acid contents, Vanderplanck et al., 2014; toxic substances in nectar, Carey, Lewis, MacGregor, & Martin‐Smith, 1959) and the scarcity of available alternative resources could explain why insects mostly foraged outside high‐density patches. Third, some pollinators (*Sphaerophoria batava*,*S. scripta*, *Empis euempis tessellata*) carried no or very little *R. ferrugineum* pollen (*Volucella bombylans*, *Empis leptempis pandellei*, *Bombus wurflenii*) and appeared to reluctantly forage on the shrub which was consequently very underrepresented in networks compared to its abundance in communities. Even the main visitors, *Apis mellifera* and *Bombus lucorum*, were far from only visiting *R. ferrugineum*, but visited even more species in high‐density patches than in low density ones. These results are consistent with previous findings showing (a) that Diptera usually prefer simpler shaped actinomorphic flowers with exposed rewards (Pornon et al., 2016; Stanley & Stout, 2014) whereas there could be a trait mismatch between the tubular weakly zygomorphic *R. ferrugineum* corolla and the fly's buccal apparatus, (b) a decrease in pollinator density (mostly Diptera) on *R. ferrugineum* and an increase in the surrounding community with an increase in shrub density (Delmas et al., 2014). Our results highlight the fact that a native mass‐flowering plant may play a less central role and be less dominant in networks than crops or supergeneralist alien plants (Lopezaraiza‐Mikel et al., 2007; Magrach et al., 2017; Stanley & Stout, 2014; Tiedeken & Stout, 2015; Vilà et al., 2011). However, more studies are required to assess the extent to which our findings can be generalized to other mass‐flowering species. Long‐term evolutionary and filtering mechanisms assembling complementary plant species and/or species developing positive interactions (Ghazoul, 2006) could have prevented the mass‐flowering plant from monopolizing pollinators at the expense of coflowering species. Indeed, pollinator monopolization could be detrimental to the survival of coflowering plant populations and, over time, to the survival of the mass‐flowering plant itself, as a single species would not be able to support a diversified, abundant, and functional pollinator community on its own.

### 4.2 The increase in insect specialization in low‐density patches

Individual and species pollinators generally tended to reduce generally their niche breadth and have higher species diet segregation in response to the reduced floral displays (Bolnick et al., 2003). In response to the decrease in local resource availability, individual *Empididae* tended to extend exclusive interactions to rare plant species (higher *d'* insect) that differed from those used by their conspecifics (lower PS*_i_*), a shift likely favored by their low individual food needs. On the contrary, probably because rare plants did not provide sufficient resources for colony needs, individual *Apidae* tended to forage the same (higher PS*_i_*) relatively abundant species (lower *d'* insect) in low‐density patches. In high‐density patches, in addition to the mass‐flowering plant, individuals were able to use a wide range of rare plants (higher *d'* and lower PS*_i_*). The functional consequences of such shifts are potentially important because they suggest that rare plant species will always be visited and will sustainably coexist with widespread mass‐flowering plants. Spatiotemporal niche complementarity of pollinators is believed to be one of the main drivers of the relationship between insect diversity and pollination efficiency (Albrecht, Schmid, Hautier, & Müller, 2012; Fontaine et al., 2008). Taxonomic differences in response to floral changes may also reinforce this relationship and hence contribute to network resilience in the face of environmental changes. Although we were unable to provide direct evidence for competition, its involvement in the observed pattern is highly probable. Indeed, the decrease in pollinator abundance from high‐density to low‐density patches was much lower than that of floral availability. Consequently, at the same site, Delmas et al. (2014) found an increase in pollinator density on isolated *R. ferrugineum* shrubs and smaller insect pollen loads, suggesting stronger competition for pollen and possibly for nectar rewards. Furthermore, the fact that *Polygala calcarea* was not visited and therefore could not compensate for the decrease in the mass‐flowering plant likely increased the exploitative competition in the low‐density patch LDP1. The lower degree of linkage and the higher dietary segregation of insect species in low‐density patches are consistent with the widely shared view that coexisting species diverge in resource use to mitigate the consequences of interspecific competition (Araújo et al., 2011; Inouye, 1978; Van Valen, 1965). Therefore, the observed pattern could represent the net ultimate balance between the diversifying effect (individual specialization in various plant species) of intraspecific competition and the constraints in interspecific competition on species generalization (Bolnick et al., 2003; Roughgarden, 1972).

### 4.3 Paradox and complementarity in pollination networks

The different approaches we used in this study reveal a quite paradoxical view of pollination and a functional complementarity into networks. On the one hand, consistent with previous findings (Banza, Belo, & Evans, 2015; Lucas et al., 2018; Tur, Vigalondo, Trøjelsgarrd, Olensen, & Traveset, 2014) our observations based on surveys of the occurrence of links revealed that individuals and particularly species of pollinators had large food niches (high *L_i_* and *L*_sp_); that is, they interacted with many plant species. Individual (*i*‐sp N_link_) and species (sp‐sp N_link_) networks were therefore also highly generalized, regardless of *R. ferrugineum* density. Consistently, the WIC/TNW ratio (0.53) was higher than or comparable with ratio reported for other generalized systems (Bolnick et al., 2003, 0.47 and 0.54; Tur et al., 2014, 0.43 and 0.48) and only slightly lower than the average calculated for a broad array of taxa (Araújo et al., 2011; 0.66). On the other hand, food niche indices based on link strength (*L*_sp (80%)_, *L_i _*_(80%)_) revealed that individuals and overall species used plant species very unevenly. Thus, paradoxically, generalist individuals behaved like specialists foraging on a few very specific plant species. Even generalist species characterized by a large diet breadth foraged intensively on a small number of plant species. Consequently, networks using sequence counts (*i*‐sp N_seq_ and sp‐sp N_seq_) were clearly much more specialized than networks only based on links (*i*‐sp N_link_ and sp‐sp N_link_). Furthermore, the low species (SPO) and individual (IO) niche overlaps, together with the relatively low proportional individual‐species diet similarity, demonstrated high niche segregation. Thus, pollination networks appeared to be more complementary than redundant.

The tendency of generalist species to be highly specialized at the individual level is certainly a crucial characteristic of the functioning of pollination networks. Indeed, a large diet breadth may allow individuals to use alternative resources in response to spatiotemporal changes in floral availability. Specialization and complementarity may help reduce interindividual and interspecific exploitative competition (Bolnick et al., 2003) for floral resources and may thus increase population stability and favor species coexistence in pollinator assemblages, especially in a context of limited or changing floral resources. However, high complementarity (low redundancy) may undermine networks in the context of global changes through cascading extinctions (Memmott et al., 2004). Specialization benefits plants by enhancing conspecific pollen transfer and reducing the loss of pollen during interspecific flights (Morales & Traveset, 2008), thus increasing the fitness and population dynamics of plants. Together, these mechanisms may help ensure the stability of pollination networks.

### 4.4 Potential limits of metabarcoding

There is now enough evidence that metabarcoding is a reliable way to characterize species composition in environmental DNA samples (Ji et al., 2013) including pollen samples (Bell et al., 2018). Further, several studies have highlighted the potential of metabarcoding for quantification of the abundance of plant species in pollen mixtures especially with plastid markers (Kraaijeveld et al., 2015; Richardson et al., 2019), whereas other markers (most often ITS2, not used in our study) were apparently less efficient (Bell et al., 2018; Richardson et al., 2019). Nonetheless, some potential biases (Deiner et al., 2017) may alter quantification accuracy. First, a plant species–molecular marker interaction is often observed during PCR, so that a species may be properly amplified by one marker but not by another one. Such amplification failures may lead to underrepresentation of the species concerned in sequencing products and to subsequent proportional over‐representation of the other species (Bell et al., 2018), thus increasing specialization level in networks. In our study, the risk of species underrepresentation (and hence the risk of proportional over‐representation of other species) was limited since we combined two markers and, for every plant species, only kept the results of the marker whose amplification was the most successful (Pornon et al., 2016). Second, the composition and relative abundance of species in multispecies pollen mixtures may also hinder quantification (Bell et al., 2018) but, at least with our protocol, only partially (Pornon et al., 2016). Third, authors who tested metabarcoding quantification used natural (bee corbicula) or artificial mixtures with huge pollen quantities (Bell et al., 2018; Cornman et al., 2015; Galimberti et al., 2014; Keller et al., 2015; Richardson et al., 2019; Smart et al., 2017), which we did not. High pollen quantities may increase concentrations of endogenous inhibitors during PCR due, for instance, to high CG contents in ITS sequences (Mammedov et al., 2008). Fourth, the abundance of plant species in insect pollen loads could to some extent, depend on the rate of plant‐to‐insect pollen transfers per visit. Therefore, despite the precautions we took, we cannot totally rule out the impact of biological/manipulation biases in our study. However, using the same raw data set as in the present study, we previously found highly significant positive correlations between the number of insect visits to a plant species and the number of its sequences in sequencing products (Pornon et al., 2016). This was the case despite the fact that visits to flowers does not translate always into pollen transport by insects (Popic, Wardle, & Davila, 2013). Finally, as long as the above biases remained consistent across floral patches (we cannot see why this should not be the case) they could not be responsible for the higher pollinator specialization in low‐density patches than in high‐density patches.

## CONFLICT OF INTEREST

None declared.

## AUTHOR'S CONTRIBUTIONS

AP supervised the study. AP, NE, MB, and CA collected the data; NE and SB performed laboratory works. CA, SB, and AP analyzed the data. AP led the writing of the manuscript. All authors contributed critically to the drafts and gave their final approval for publication.

## Data Availability

Nucleotide sequences have been published on GenBank (Accession numbers KU974005‐KU974022, KU974024‐KU974083).
