# Supplementary material for: Pollinator specialization increases with a decrease in a mass‐flowering plant in networks inferred from DNA metabarcoding
Source: Ecol Evol. 2019 Sep 30;9(24):13650–62. doi: 10.1002/ece3.5531 (PMC6953672; doi:10.1002/ece3.5531)

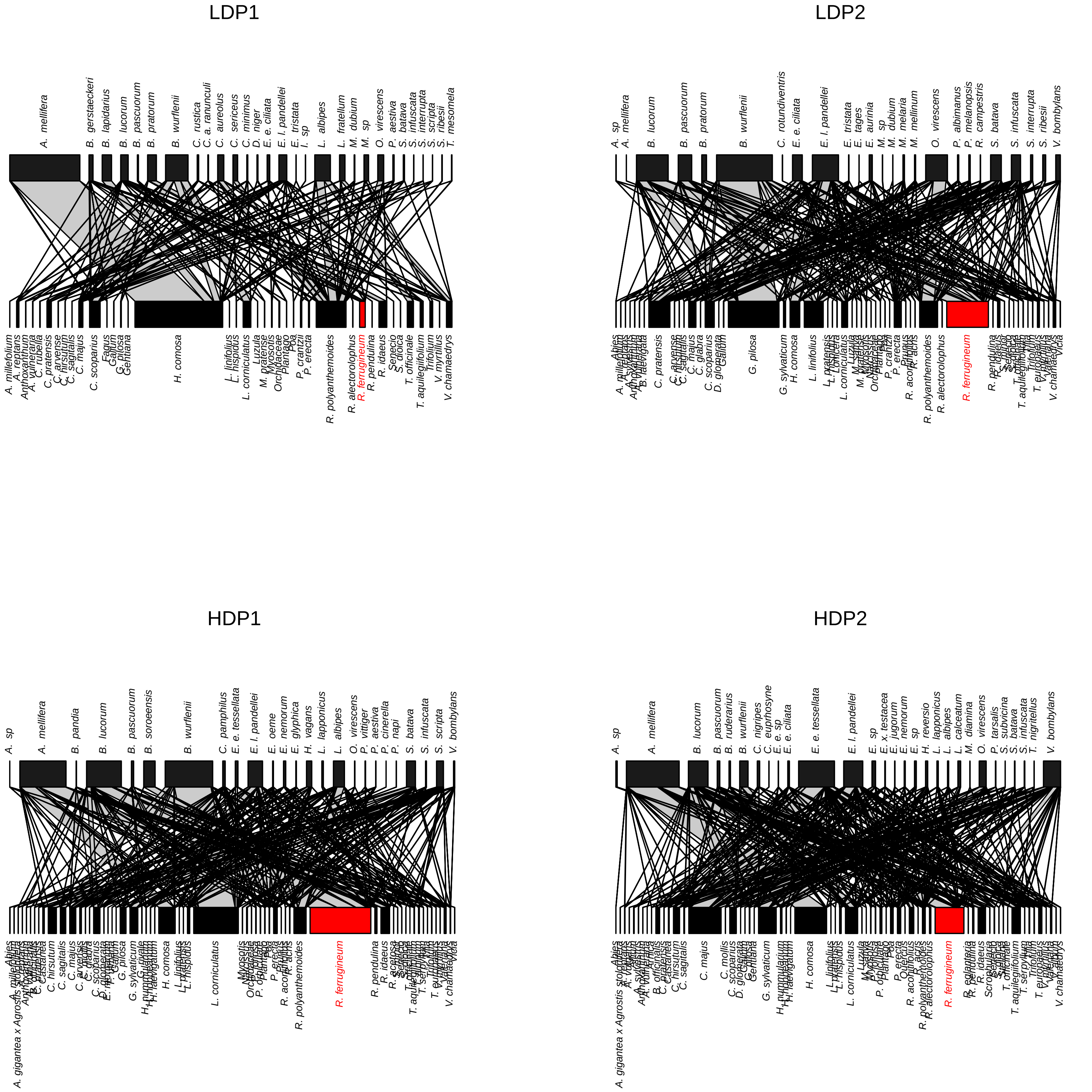
LDP1
LDP2
HDP1
HDP2
A. mellifera
B. gerstaeckeri
B. lapidarius
B. lucorum
B. pascuorum
B. pratorum
B. wurflenii
C. rustica
C. a. ranunculi
C. aureolus
C. sericeus
C. minimus
D. niger
E. e. ciliata
E. l. pandellei
E. tristata
I. sp
L. albipes
L. fratellum
M. dubium
M. sp
O. virescens
P. aestiva
S. batava
S. infuscata
S. interrupta
S. scripta
S. ribesii
T. mesomela
H. comosa
R. polyanthemoides
R. ferrugineum
L. corniculatus
G. pilosa

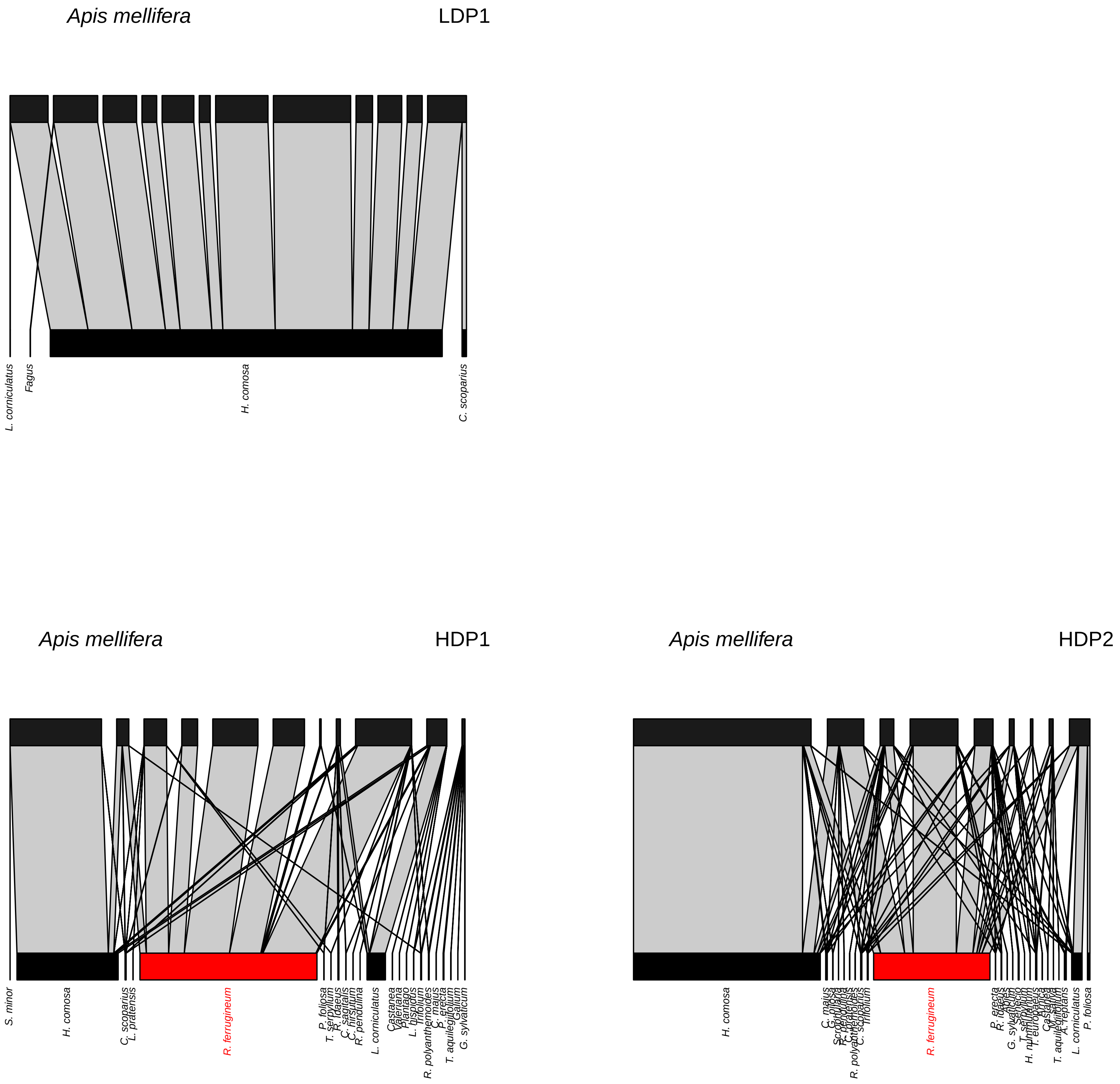
Apis mellifera
LDP1
L. corniculatus
Fagus
H. comosa
C. scoparius
Apis mellifera
HDP1
S. minor
H. comosa
C. scoparius
L. pratensis
R. ferrugineum
P. foliosa
T. serpyllum
R. idaeus
C. sagittalis
C. hirsutum
R. pendulina
L. corniculatus
Castanea
Valeriana
Plantago
L. hispidus
Trifolium
R. polyanthemoides
C. majus
P. erecta
T. aquilegiifolium
Galium
G. sylvaticum
Apis mellifera
HDP2
H. comosa
C. majus
R. polyanthemoides
C. scoparius
Trifolium
R. ferrugineum
P. erecta
R. idaeus
T. aquilegiifolium
A. reptans
L. corniculatus
P. foliosa

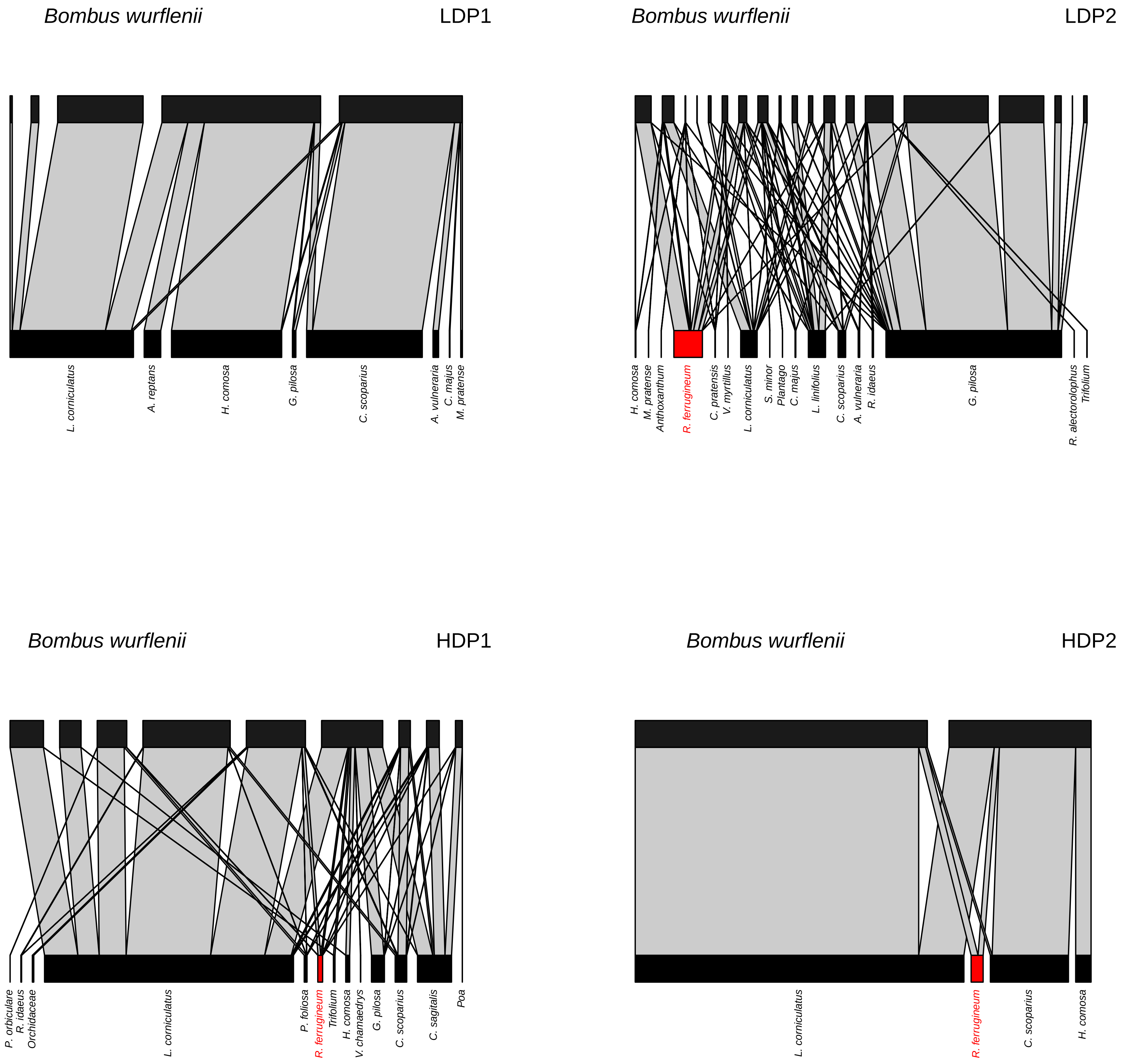
Bombus wurflenii
LDP1
L. corniculatus
A. reptans
H. comosa
G. pilosa
C. scoparius
A. vulneraria
C. majus
M. pratense
Bombus wurflenii
LDP2
H. comosa
M. pratense
Anthoxanthum
R. ferrugineum
C. pratensis
V. myrtillus
L. corniculatus
S. minor
Plantago
C. majus
L. linifolius
C. scoparius
A. vulneraria
R. idaeus
G. pilosa
R. alectorolophus
Trifolium
Bombus wurflenii
HDP1
P. orbiculare
R. idaeus
Orchidaceae
L. corniculatus
P. foliosa
R. ferrugineum
Trifolium
H. comosa
V. chamaedrys
G. pilosa
C. scoparius
C. sagitalis
Poa
Bombus wurflenii
HDP2
L. corniculatus
R. ferrugineum
C. scoparius
H. comosa

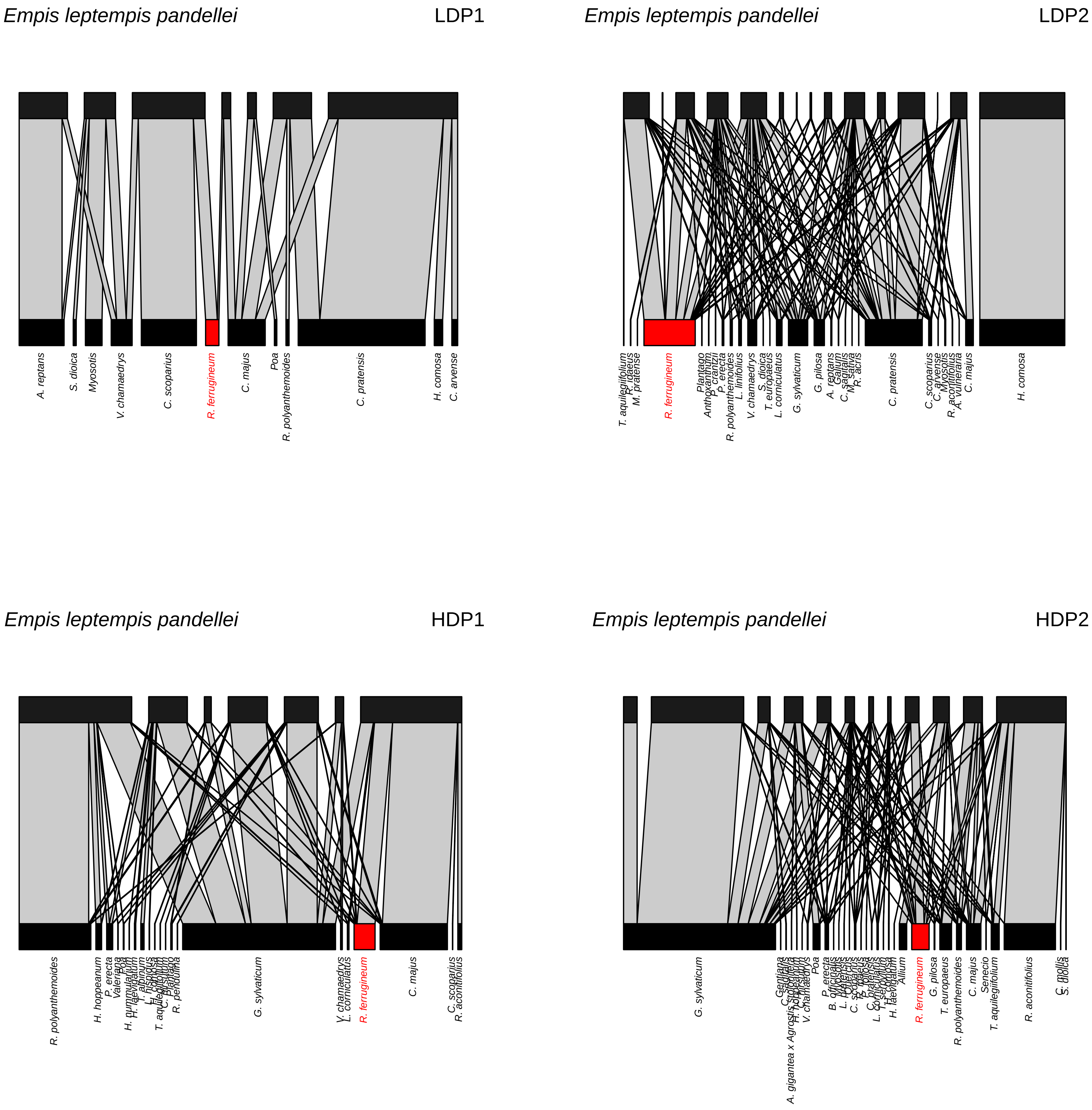
Empis leptempis pandellei
LDP1
A. reptans
S. dioica
Myosotis
V. chamaedrys
C. scoparius
R. ferrugineum
C. majus
Poa
R. polyanthemoides
C. pratensis
H. comosa
C. arvense
Empis leptempis pandellei
LDP2
T. aquilegiifolium
R. idaeus
M. pratense
R. ferrugineum
Plantago
Anthoxanthum
P. crantzii
P. erecta
R. polyanthemoides
L. linifolius
V. chamaedrys
S. dioica
T. europaeus
L. corniculatus
G. sylvaticum
G. pilosa
A. reptans
Galium
C. sagittalis
R. acris
C. pratensis
C. scoparius
C. arvense
Myosotis
R. aconitifolius
A. vulneraria
C. majus
H. comosa
Empis leptempis pandellei
HDP1
R. polyanthemoides
H. hoppeanum
P. erecta
Valeriana
H. nummularium
R. pendulina
G. sylvaticum
V. chamaedrys
L. corniculatus
R. ferrugineum
C. majus
C. scoparius
R. aconitifolius
Empis leptempis pandellei
HDP2
G. sylvaticum
Gentiana
A. gigantea x Agrostis
V. chamaedrys
Poa
P. erecta
B. officinalis
C. scoparius
L. corniculatus
H. laevigatum
Allium
R. ferrugineum
G. pilosa
T. europaeus
R. polyanthemoides
C. majus
Senecio
T. aquilegiifolium
R. aconitifolius
C. mollis
S. dioica

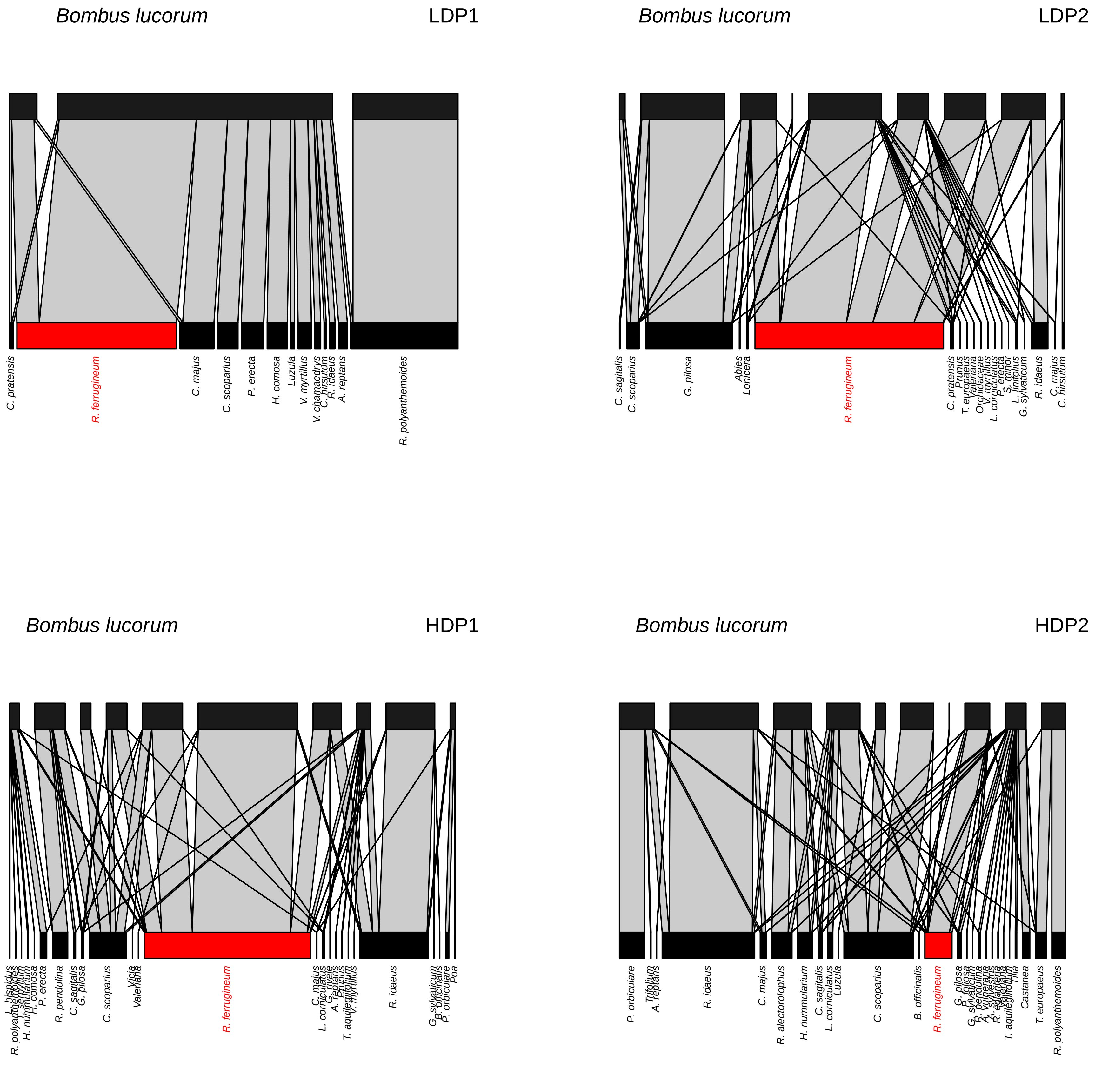
Bombus lucorum
LDP1
C. pratensis
R. ferrugineum
C. majus
C. scoparius
P. erecta
H. comosa
Luzula
V. myrtillus
V. chamaedrys
C. hirsutum
R. idaeus
A. reptans
R. polyanthemoides
Bombus lucorum
LDP2
C. sagitalis
C. scoparius
G. pilosa
Abies
Lonicera
R. ferrugineum
C. pratensis
Prunus
T. europaeus
Valeriana
Orchidaceae
V. myrtillus
L. corniculatus
P. erecta
S. minor
L. linifolius
G. sylvaticum
R. idaeus
C. majus
C. hirsutum
Bombus lucorum
HDP1
L. hispidus
R. polyanthemoides
H. nummularium
H. comosa
P. erecta
R. pendulina
C. sagitalis
G. pilosa
C. scoparius
Vicia
Valeriana
R. ferrugineum
C. majus
L. corniculatus
A. reptans
Prunus
T. aquilegiifolium
V. myrtillus
R. idaeus
G. sylvaticum
B. officinalis
P. orbiculare
Poa
Bombus lucorum
HDP2
P. orbiculare
Trifolium
A. reptans
R. idaeus
C. majus
R. alectorolophus
H. nummularium
C. sagitalis
L. corniculatus
Luzula
C. scoparius
B. officinalis
R. ferrugineum
G. pilosa
G. sylvaticum
R. pendulina
Valeriana
T. aquilegiifolium
Tilia
Castanea
T. europaeus
R. polyanthemoides

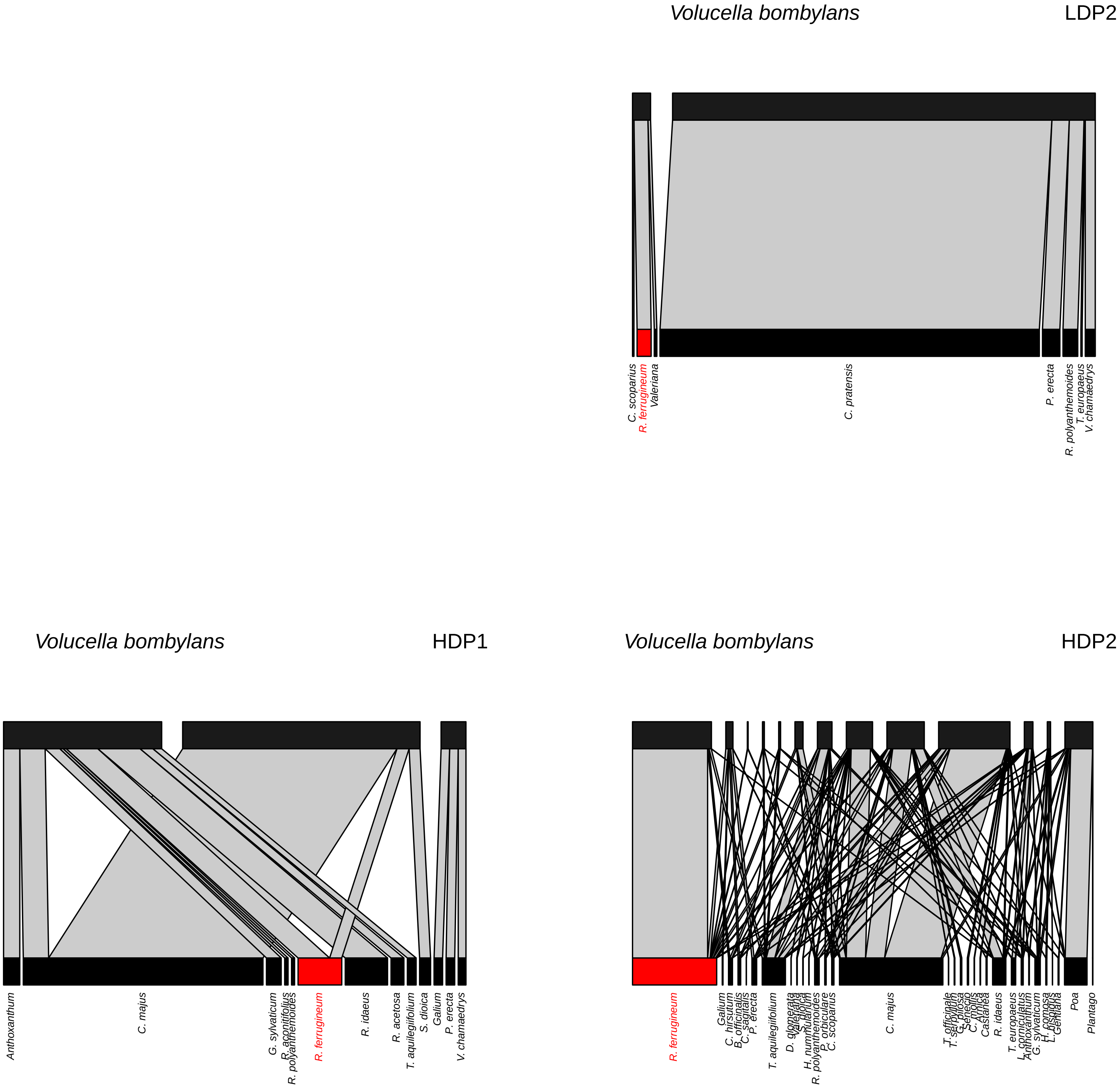
Volucella bombylans
LDP2
C. scoparius
R. ferrugineum
Valeriana
C. pratensis
P. erecta
R. polyanthemoides
T. europaeus
V. chamaedrys
Volucella bombylans
HDP1
Anthoxanthum
C. majus
G. sylvaticum
R. aconitifolius
R. polyanthemoides
R. ferrugineum
R. idaeus
R. acetosa
T. aquilegiifolium
S. dioica
Galium
P. erecta
V. chamaedrys
Volucella bombylans
HDP2
R. ferrugineum
Galium
C. hirsutum
B. officinalis
C. sagitalis
P. erecta
T. aquilegiifolium
D. glomerata
Valeriana
S. dioica
H. nummularium
R. polyanthemoides
P. orbiculare
C. scoparius
C. majus
T. officinale
T. serpyllum
Senecio
C. mollis
Arnica
Castanea
R. idaeus
T. europaeus
L. corniculatus
Anthoxanthum
G. sylvaticum
H. comosa
Gentiana
Poa
Plantago

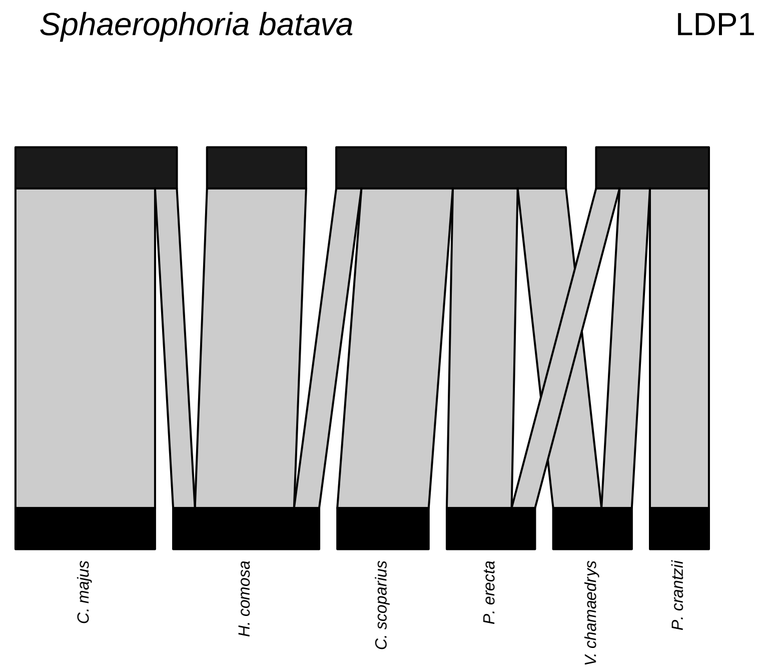
Sphaerophoria batava
LDP1
C. majus
H. comosa
C. scoparius
P. erecta
V. chamaedrys
P. crantzii

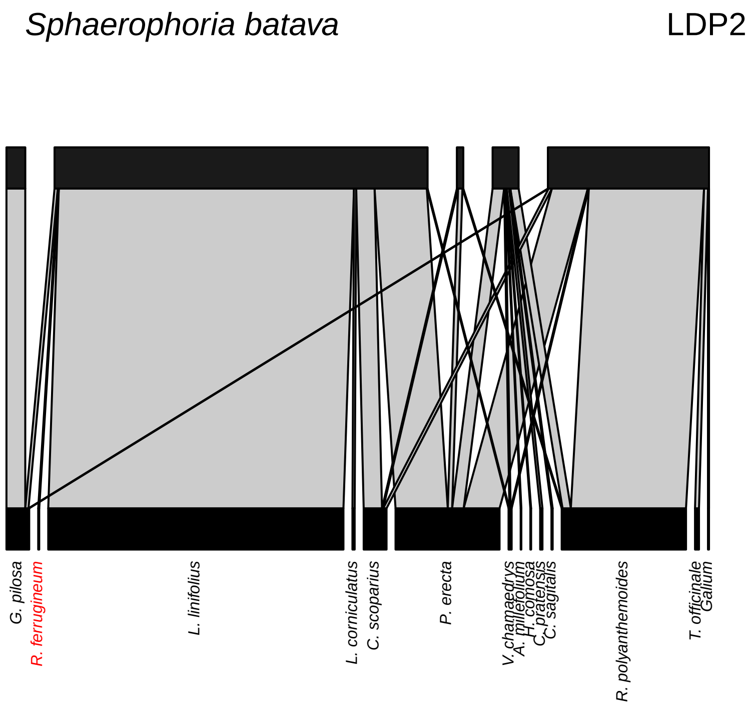
Sphaerophoria batava
LDP2
G. pilosa
R. ferrugineum
L. linifolius
L. corniculatus
C. scoparius
P. erecta
V. chamaedrys
A. millefolium
H. comosa
C. pratensis
C. sagitalis
R. polyanthemoides
T. officinale
Galium

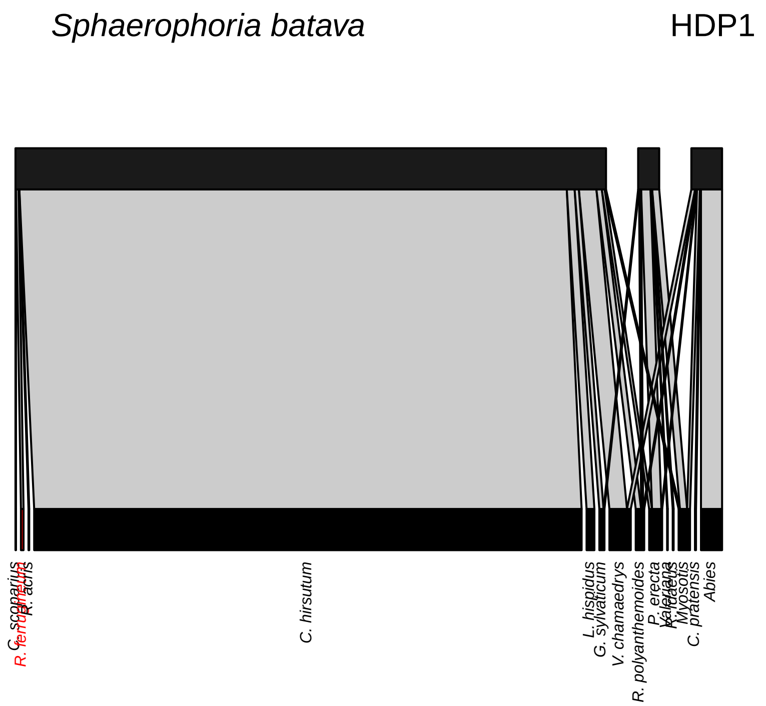
Sphaerophoria batava
HDP1
C. scoparius
R. ferrugineum
R. acris
C. hirsutum
L. hispidus
G. sylvaticum
V. chamaedrys
R. polyanthemoides
P. erecta
Valeriana
R. idaeus
Myosotis
C. pratensis
Abies

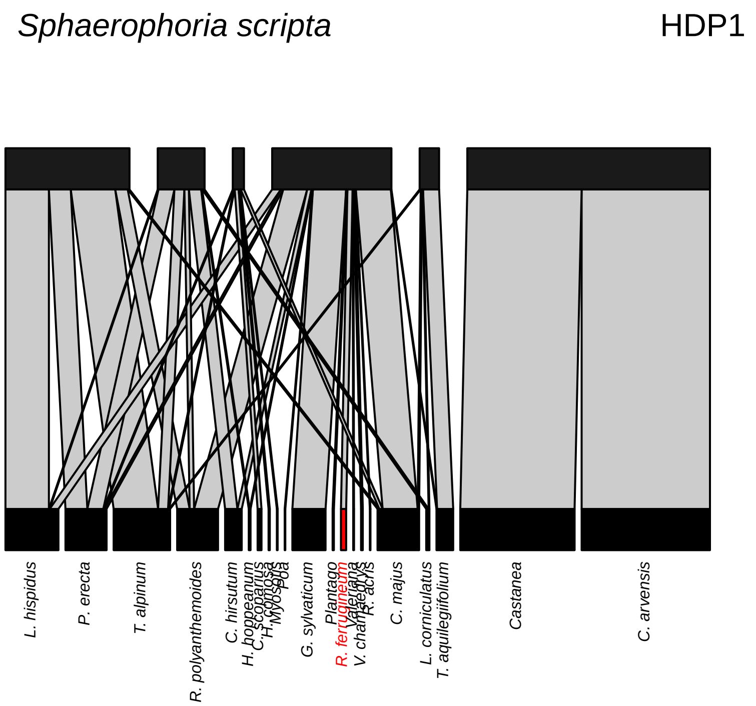

Sphaerophoria scripta
HDP1
L. hispidus
P. erecta
T. alpinum
R. polyanthemoides
C. hirsutum
G. sylvaticum
Plantago
R. ferrugineum
C. majus
L. corniculatus
T. aquilegiifolium
Castanea
C. arvensis

Empis euempis tessellata HDP2

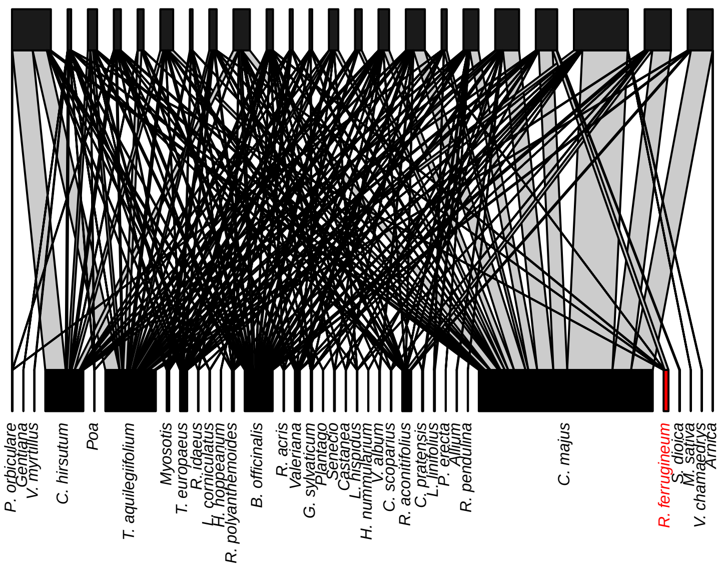

Sphaerophoria interrupta LDP1

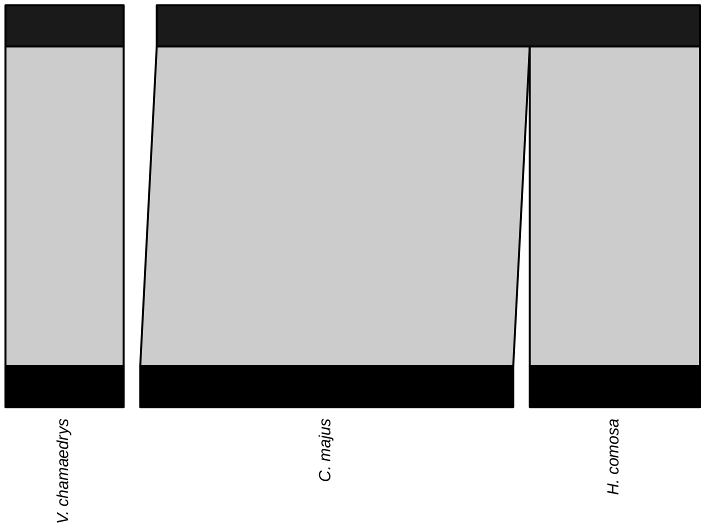

Empis euempis ciliata LDP2

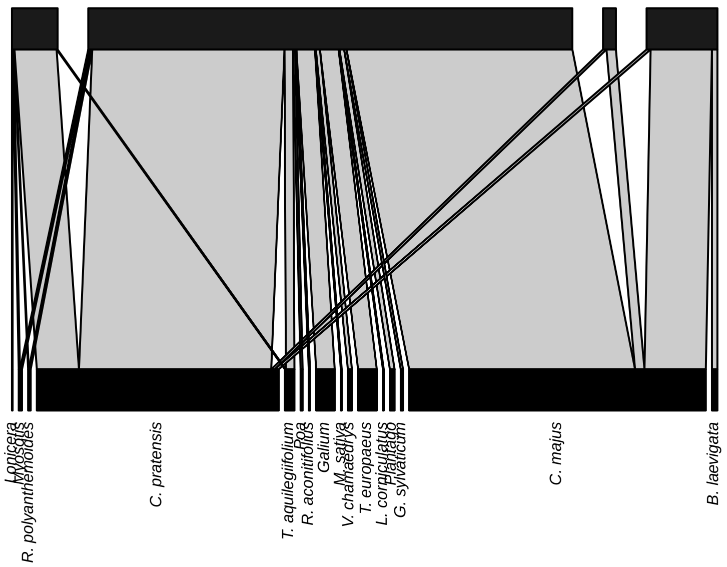

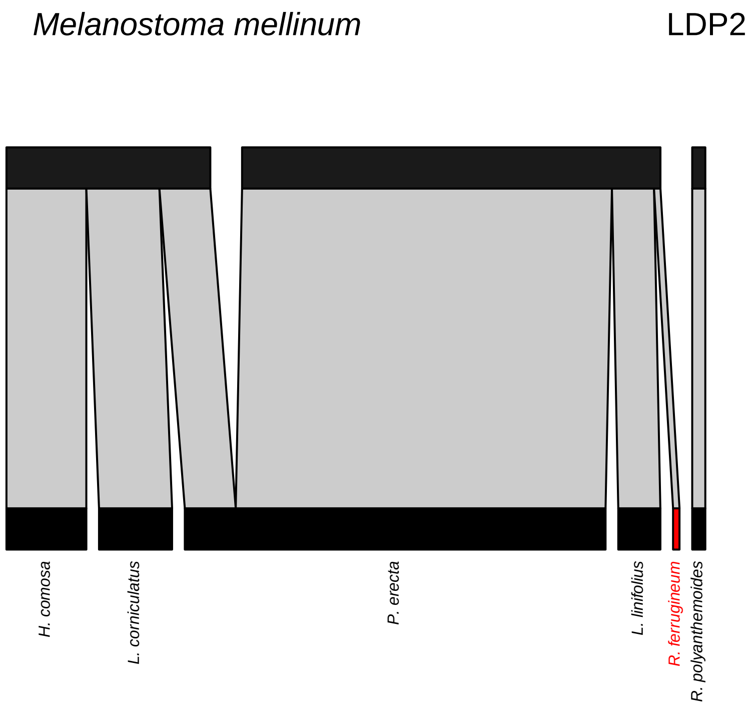
Melanostoma mellinum
LDP2
H. comosa
L. corniculatus
P. erecta
L. linifolius
R. ferrugineum
R. polyanthemoides

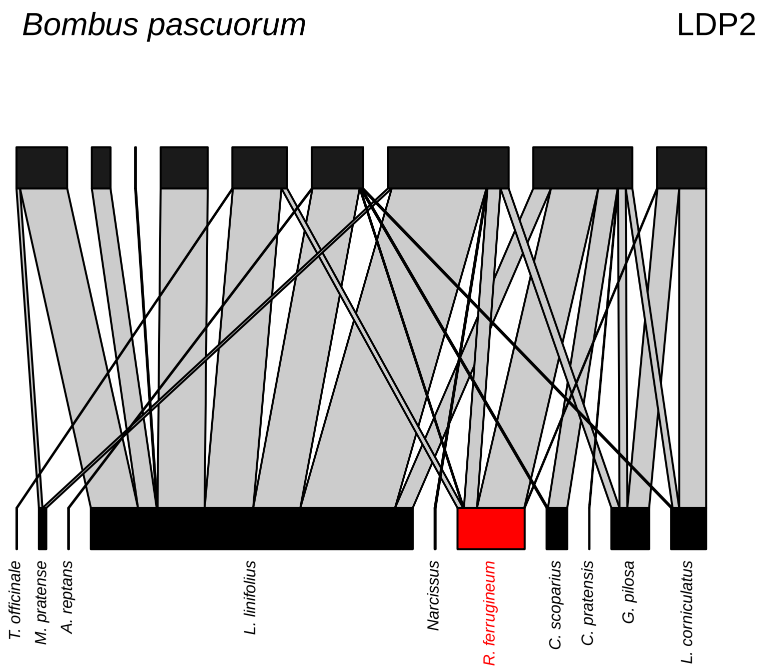

Bombus pascuorum
LDP2
T. officinale
M. pratense
A. reptans
L. linifolius
Narcissus
R. ferrugineum
C. scoparius
C. pratensis
G. pilosa
L. corniculatus

Bombus pratorum LDP2

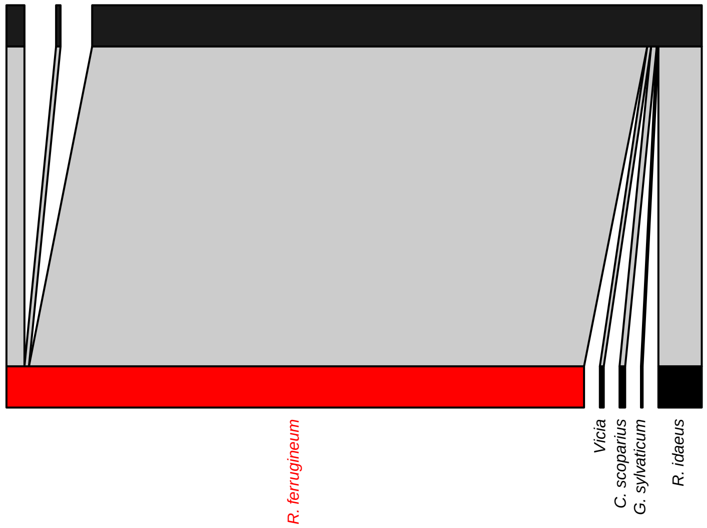

Sphaerophoria infuscata LDP2

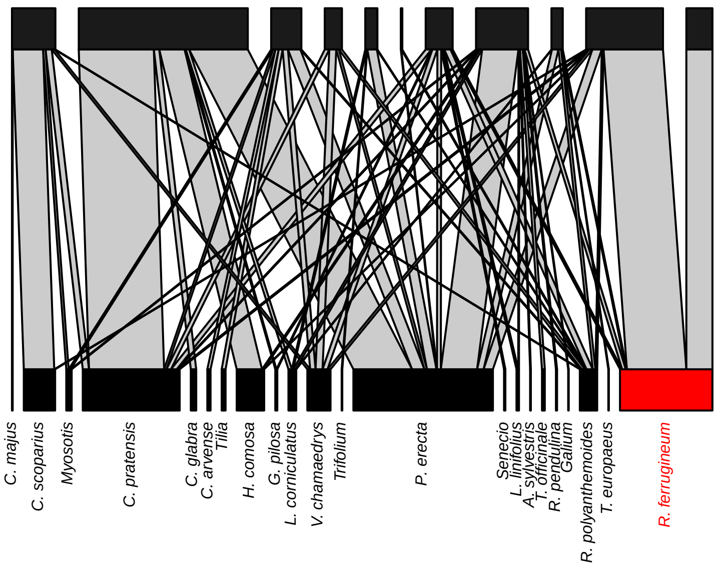

Supplement: Supplementary file 1 [file ECE3-9-13650-s001.pdf]
