# Supplementary material for: Pollinator specialization increases with a decrease in a mass‐flowering plant in networks inferred from DNA metabarcoding
Source: Ecol Evol. 2019 Sep 30;9(24):13650–62. doi: 10.1002/ece3.5531 (PMC6953672; doi:10.1002/ece3.5531)
Supplement: Supplementary file 2 [file ECE3-9-13650-s002.pdf]

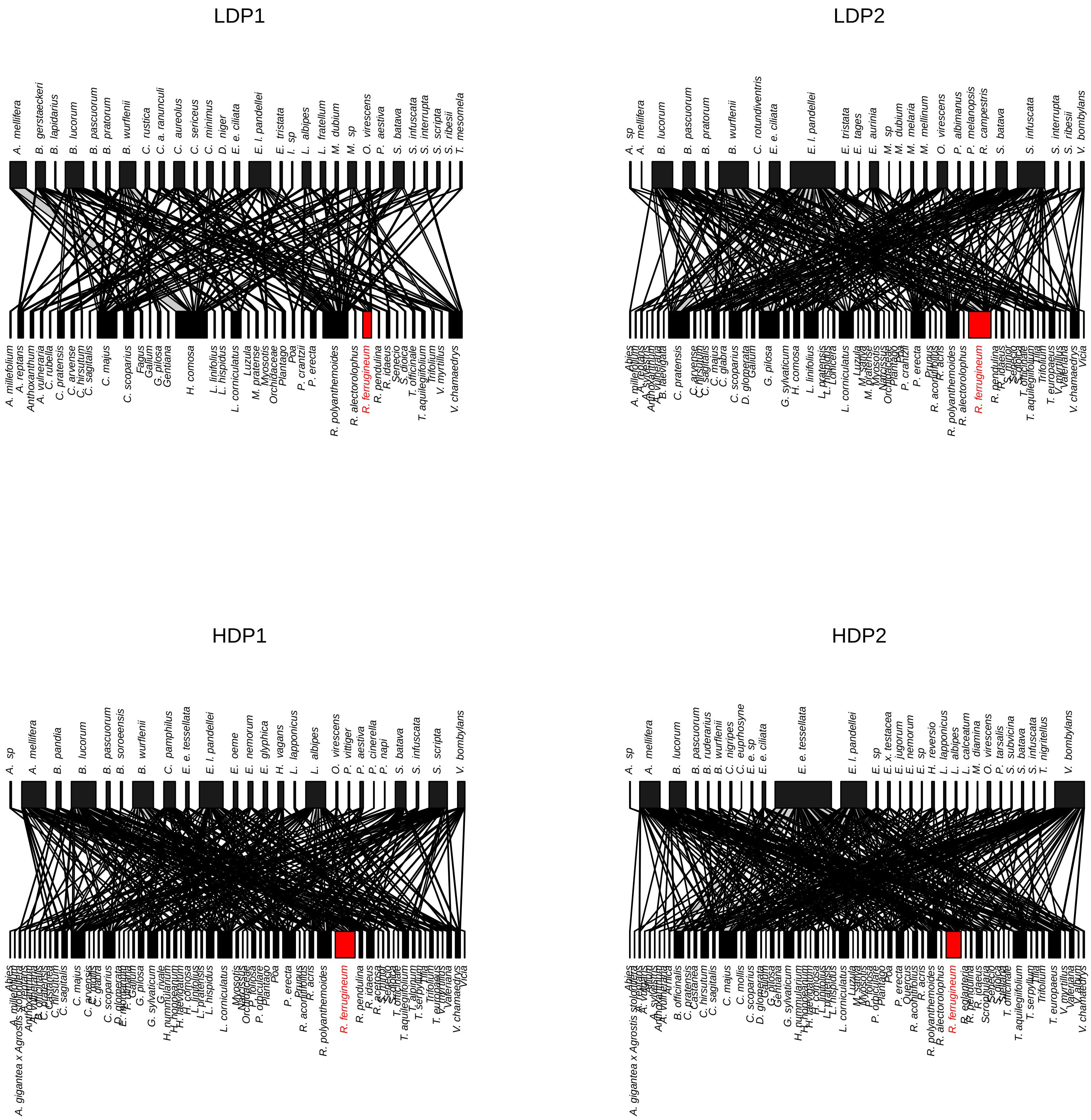
LDP1
A. mellifera
B. gerstaeckeri
B. lapidarius
B. lucorum
B. pascuorum
B. pratorum
B. wurflenii
C. rustica
C. a. ranunculi
C. aureolus
C. sericeus
C. minimus
D. niger
E. e. ciliata
E. l. pandellei
E. tristata
I. sp
L. albipes
L. fratellum
M. dubium
M. sp
O. virescens
P. aestiva
S. batava
S. infuscata
S. interrupta
S. scripta
S. ribesii
T. mesomela
A. millefolium
A. reptans
Anthoxanthum
A. vulneraria
C. rubella
C. pratensis
C. arvense
C. hirsutum
C. sagitalis
C. majus
C. scoparius
Fagus
Galium
G. pilosa
Gentiana
H. comosa
L. linifolius
L. hispidus
L. corniculatus
Luzula
M. pratense
Myosotis
Orchidaceae
Plantago
Poa
P. crantzii
P. erecta
R. polyanthemoides
R. alectorolophus
R. ferrugineum
R. pendulina
R. idaeus
Senecio
S. dioica
T. officinale
T. aquilegiifolium
Trifolium
V. myrtillus
V. chamaedrys
LDP2
A. sp
A. mellifera
B. lucorum
B. pascuorum
B. pratorum
B. wurflenii
C. rotundiventris
E. e. ciliata
E. l. pandellei
E. tristata
E. tages
E. aurinia
M. sp
M. dubium
M. melaria
M. mellinum
O. virescens
P. albimanus
P. melanopsis
R. campestris
S. batava
S. infuscata
S. interrupta
S. ribesii
V. bombylans
R. ferrugineum
HDP1
A. sp
A. mellifera
B. pandia
B. lucorum
B. pascuorum
B. soroeensis
B. wurflenii
C. pamphilus
E. e. tessellata
E. l. pandellei
E. oeme
E. nemorum
E. glyphica
H. vagans
L. lapponicus
L. albipes
O. virescens
P. vittiger
P. aestiva
P. cinerella
P. napi
S. batava
S. infuscata
S. scripta
V. bombylans
R. ferrugineum
HDP2
A. sp
A. mellifera
B. lucorum
B. pascuorum
B. ruderarius
B. wurflenii
C. nigripes
C. euphrosyne
E. e. sp
E. e. ciliata
E. e. tessellata
E. l. pandellei
E. sp
E. x. testacea
E. jugorum
E. nemorum
E. sp
H. reversio
L. lapponicus
L. albipes
L. calceatum
M. diamina
O. virescens
P. tarsalis
S. subvicina
S. batava
S. infuscata
T. nigritellus
V. bombylans
R. ferrugineum

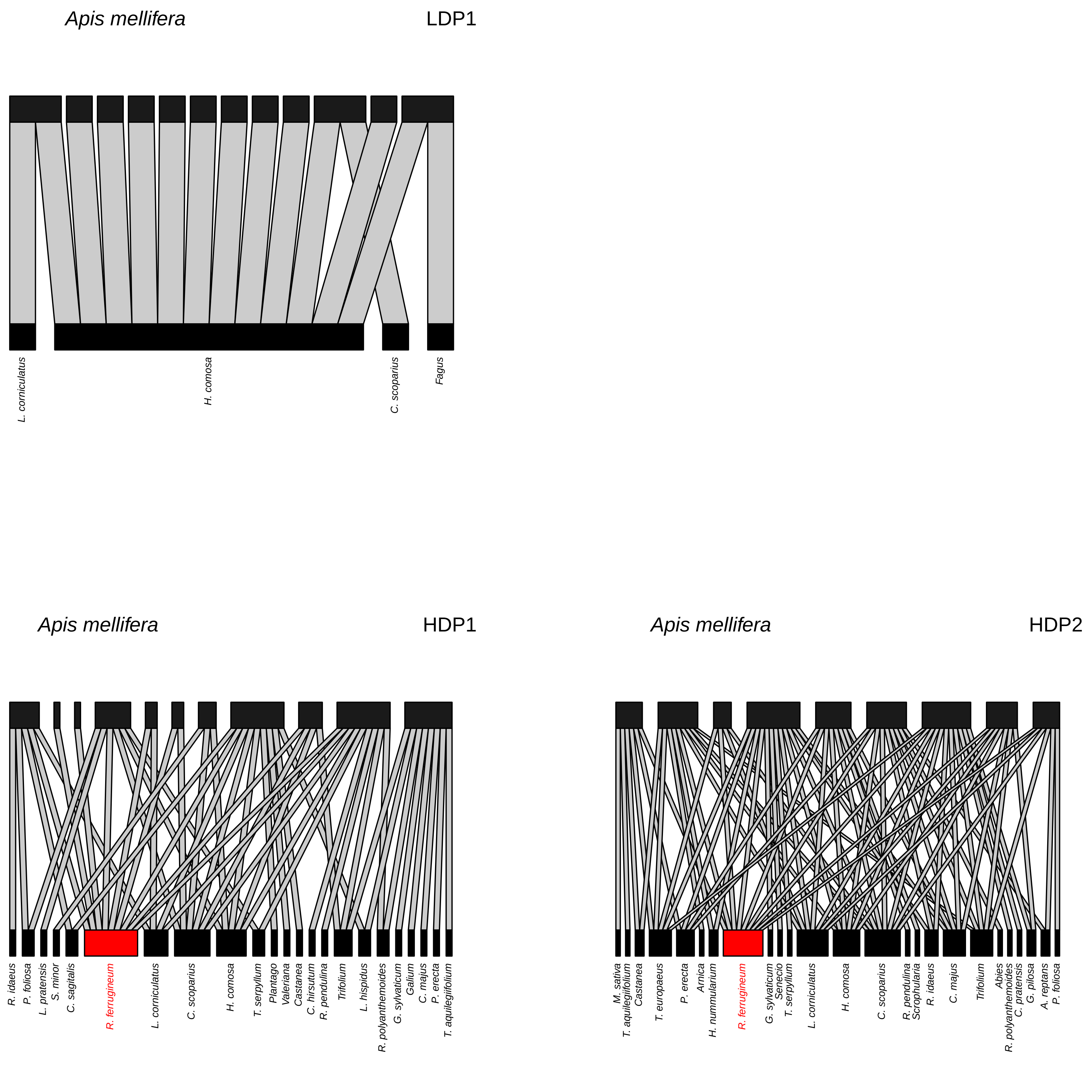

Apis mellifera
LDP1
L. corniculatus
H. comosa
C. scoparius
Fagus
Apis mellifera
HDP1
R. idaeus
P. foliosa
L. pratensis
S. minor
C. sagitalis
R. ferrugineum
L. corniculatus
C. scoparius
H. comosa
T. serpyllum
Plantago
Valeriana
Castanea
C. hirsutum
R. pendulina
Trifolium
L. hispidus
R. polyanthemoides
G. sylvaticum
Galium
C. majus
P. erecta
T. aquilegiifolium
Apis mellifera
HDP2
M. sativa
T. aquilegiifolium
Castanea
T. europaeus
P. erecta
Arnica
H. nummularium
R. ferrugineum
G. sylvaticum
Senecio
T. serpyllum
L. corniculatus
H. comosa
C. scoparius
R. pendulina
Scrophularia
R. idaeus
C. majus
Trifolium
Abies
R. polyanthemoides
C. pratensis
G. pilosa
A. reptans
P. foliosa

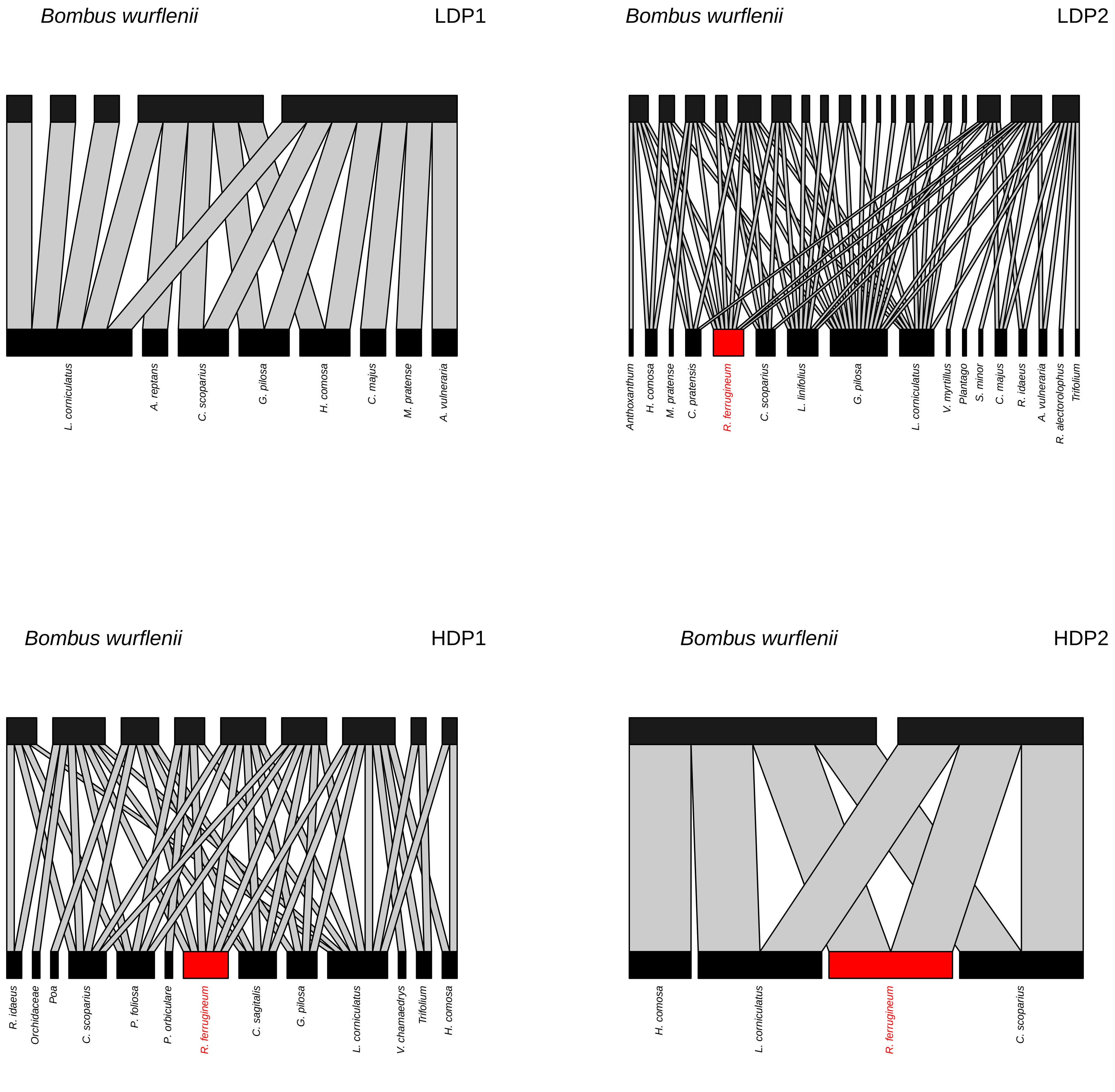
Bombus wurflenii
LDP1
L. corniculatus
A. reptans
C. scoparius
G. pilosa
H. comosa
C. majus
M. pratense
A. vulneraria
Bombus wurflenii
LDP2
Anthoxanthum
H. comosa
M. pratense
C. pratensis
R. ferrugineum
C. scoparius
L. linifolius
G. pilosa
L. corniculatus
V. myrtillus
Plantago
S. minor
C. majus
R. idaeus
A. vulneraria
R. alectorolophus
Trifolium
Bombus wurflenii
HDP1
R. idaeus
Orchidaceae
Poa
C. scoparius
P. foliosa
P. orbiculare
R. ferrugineum
C. sagitalis
G. pilosa
L. corniculatus
V. chamaedrys
Trifolium
H. comosa
Bombus wurflenii
HDP2
H. comosa
L. corniculatus
R. ferrugineum
C. scoparius

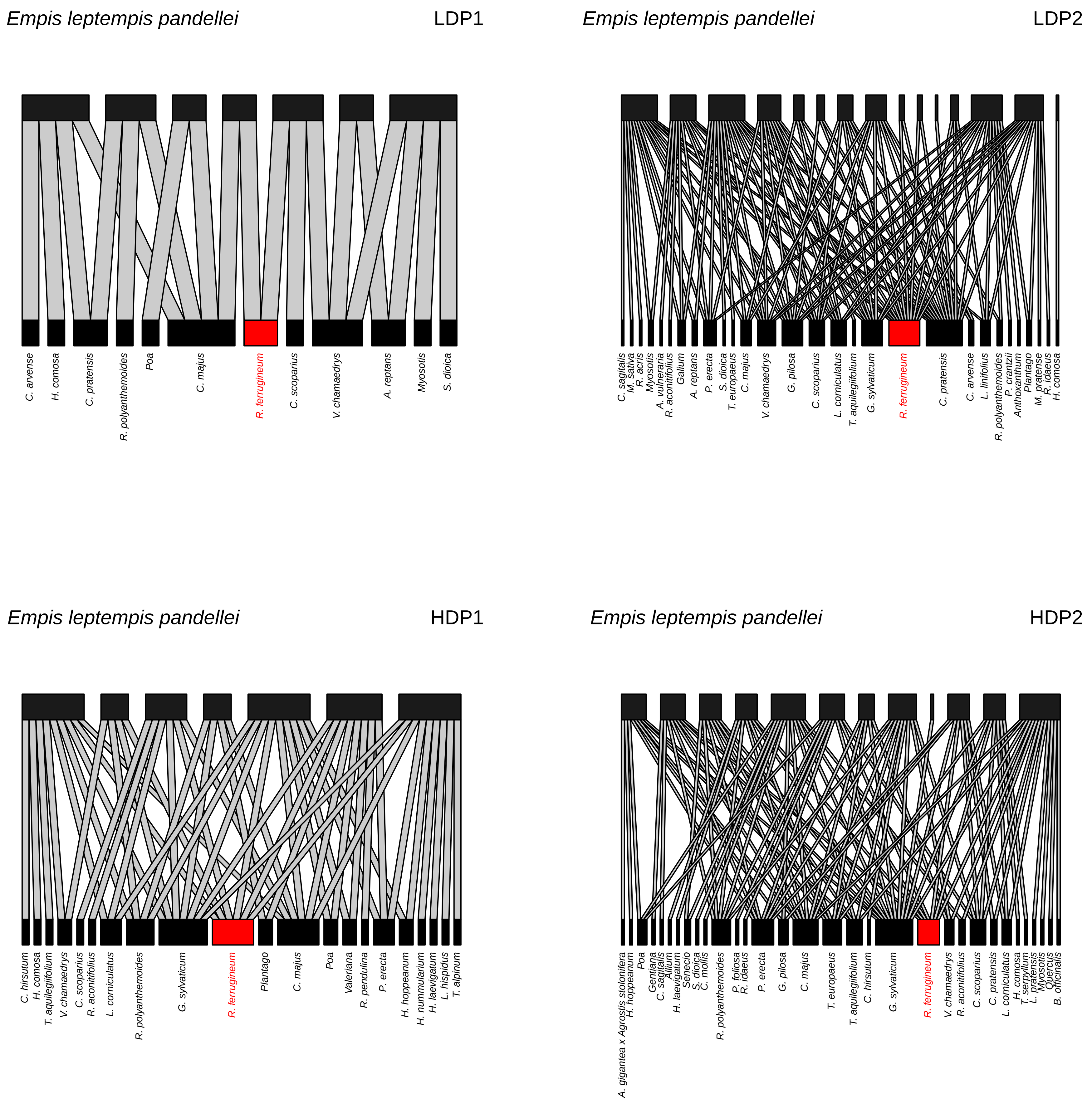

Empis leptempis pandellei
LDP1
C. arvense
H. comosa
C. pratensis
R. polyanthemoides
Poa
C. majus
R. ferrugineum
C. scoparius
V. chamaedrys
A. reptans
Myosotis
S. dioica
Empis leptempis pandellei
LDP2
C. sagitalis
M. sativa
R. acris
Myosotis
A. vulneraria
R. aconitifolius
Galium
A. reptans
P. erecta
S. dioica
T. europaeus
C. majus
V. chamaedrys
G. pilosa
C. scoparius
L. corniculatus
T. aquilegiifolium
G. sylvaticum
R. ferrugineum
C. pratensis
C. arvense
L. linifolius
R. polyanthemoides
P. crantzii
Anthoxanthum
Plantago
M. pratense
R. idaeus
H. comosa
Empis leptempis pandellei
HDP1
C. hirsutum
H. comosa
T. aquilegiifolium
V. chamaedrys
C. scoparius
R. aconitifolius
L. corniculatus
R. polyanthemoides
G. sylvaticum
R. ferrugineum
Plantago
C. majus
Poa
Valeriana
R. pendulina
P. erecta
H. hoppeanum
H. nummularium
H. laevigatum
L. hispidus
T. alpinum
Empis leptempis pandellei
HDP2
A. gigantea x Agrostis stolonifera
H. hoppeanum
Poa
Gentiana
C. sagitalis
Allium
H. laevigatum
Senecio
S. dioica
C. mollis
R. polyanthemoides
P. foliosa
R. idaeus
P. erecta
G. pilosa
C. majus
T. europaeus
T. aquilegiifolium
C. hirsutum
G. sylvaticum
R. ferrugineum
V. chamaedrys
R. aconitifolius
C. scoparius
C. pratensis
L. corniculatus
H. comosa
T. serpyllum
L. pratensis
Myosotis
Quercus
B. officinalis

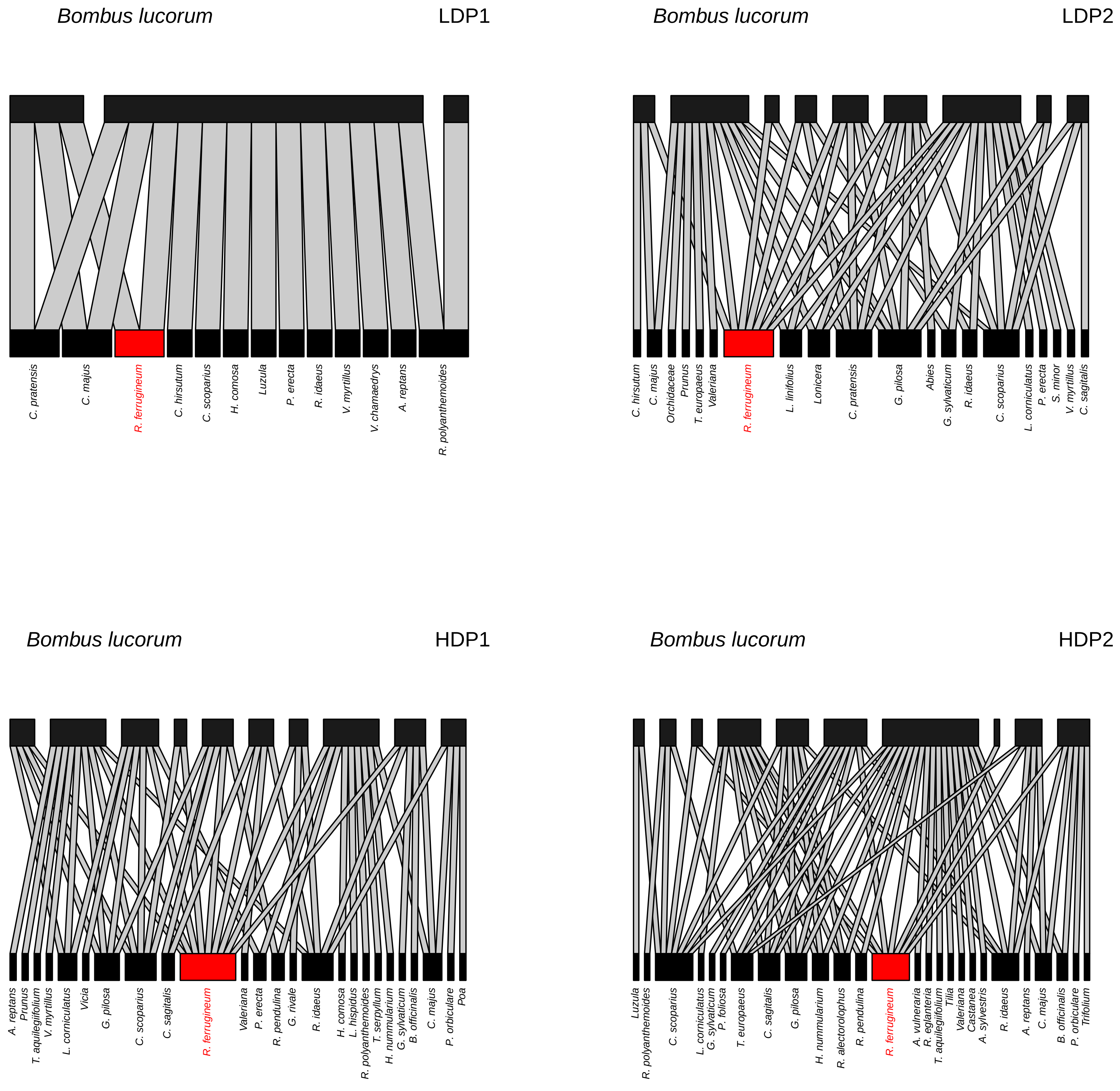
Bombus lucorum
LDP1
C. pratensis
C. majus
R. ferrugineum
C. hirsutum
C. scoparius
H. comosa
Luzula
P. erecta
R. idaeus
V. myrtillus
V. chamaedrys
A. reptans
R. polyanthemoides
Bombus lucorum
LDP2
C. hirsutum
C. majus
Orchidaceae
Prunus
T. europaeus
Valeriana
R. ferrugineum
L. linifolius
Lonicera
C. pratensis
G. pilosa
Abies
G. sylvaticum
R. idaeus
C. scoparius
L. corniculatus
P. erecta
S. minor
V. myrtillus
C. sagitalis
Bombus lucorum
HDP1
A. reptans
Prunus
T. aquilegiifolium
V. myrtillus
L. corniculatus
Vicia
G. pilosa
C. scoparius
C. sagitalis
R. ferrugineum
Valeriana
P. erecta
R. pendulina
G. rivale
R. idaeus
H. comosa
L. hispidus
R. polyanthemoides
T. serpyllum
H. nummularium
G. sylvaticum
B. officinalis
C. majus
P. orbiculare
Poa
Bombus lucorum
HDP2
Luzula
R. polyanthemoides
C. scoparius
L. corniculatus
G. sylvaticum
P. foliosa
T. europaeus
C. sagitalis
G. pilosa
H. nummularium
R. alectorolophus
R. pendulina
R. ferrugineum
A. vulneraria
R. eglanteria
T. aquilegiifolium
Tilia
Valeriana
Castanea
A. sylvestris
R. idaeus
A. reptans
C. majus
B. officinalis
P. orbiculare
Trifolium

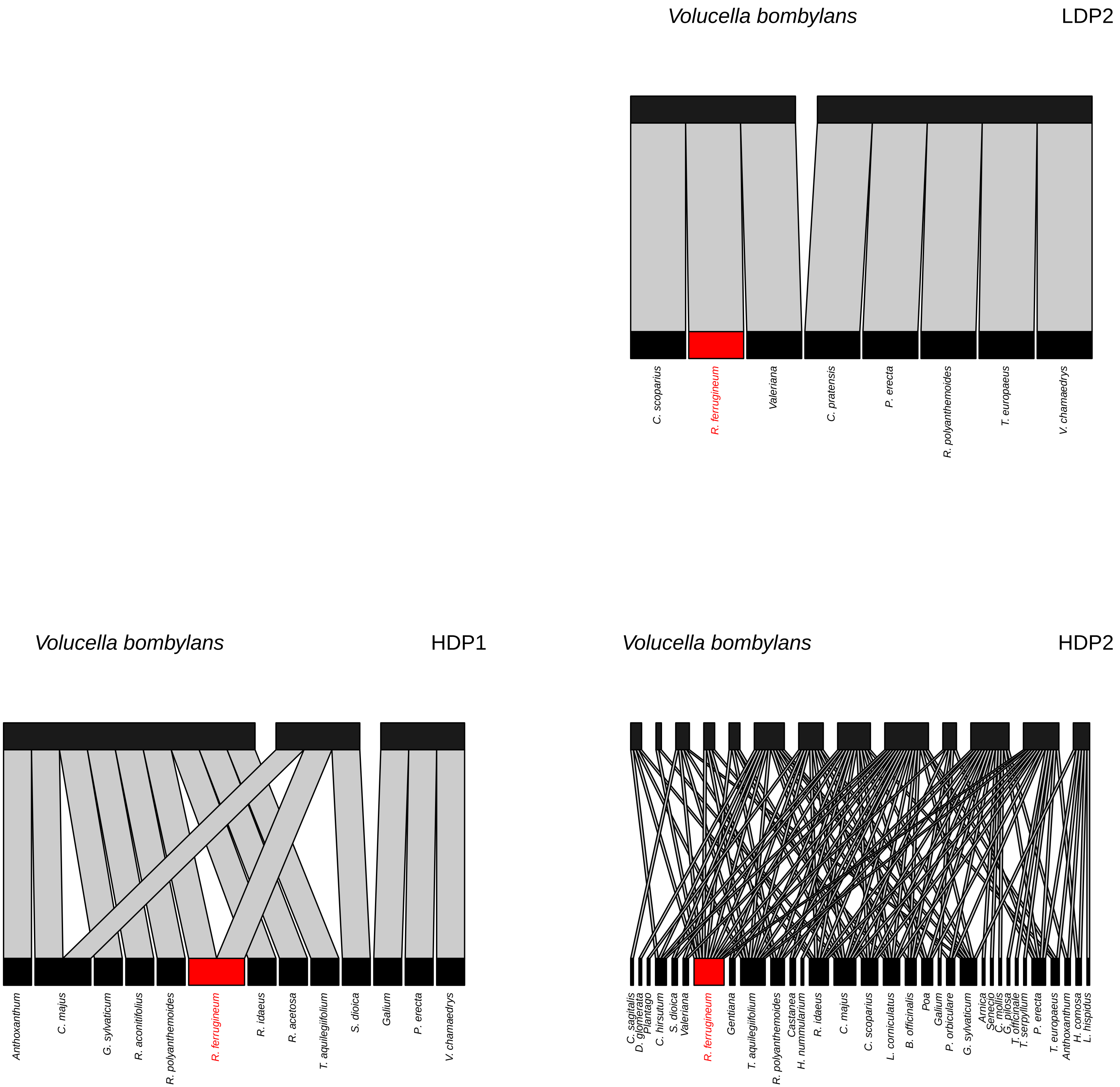
Volucella bombylans
LDP2
C. scoparius
R. ferrugineum
Valeriana
C. pratensis
P. erecta
R. polyanthemoides
T. europaeus
V. chamaedrys
Volucella bombylans
HDP1
Anthoxanthum
C. majus
G. sylvaticum
R. aconitifolius
R. polyanthemoides
R. ferrugineum
R. idaeus
R. acetosa
T. aquilegiifolium
S. dioica
Galium
P. erecta
V. chamaedrys
Volucella bombylans
HDP2
C. sagitalis
D. glomerata
Plantago
C. hirsutum
S. dioica
Valeriana
R. ferrugineum
Gentiana
T. aquilegiifolium
R. polyanthemoides
Castanea
H. nummularium
R. idaeus
C. majus
C. scoparius
L. corniculatus
B. officinalis
Poa
Galium
P. orbiculare
G. sylvaticum
Arnica
Senecio
C. mollis
G. pilosa
T. officinale
T. serpyllum
P. erecta
T. europaeus
Anthoxanthum
H. comosa
L. hispidus

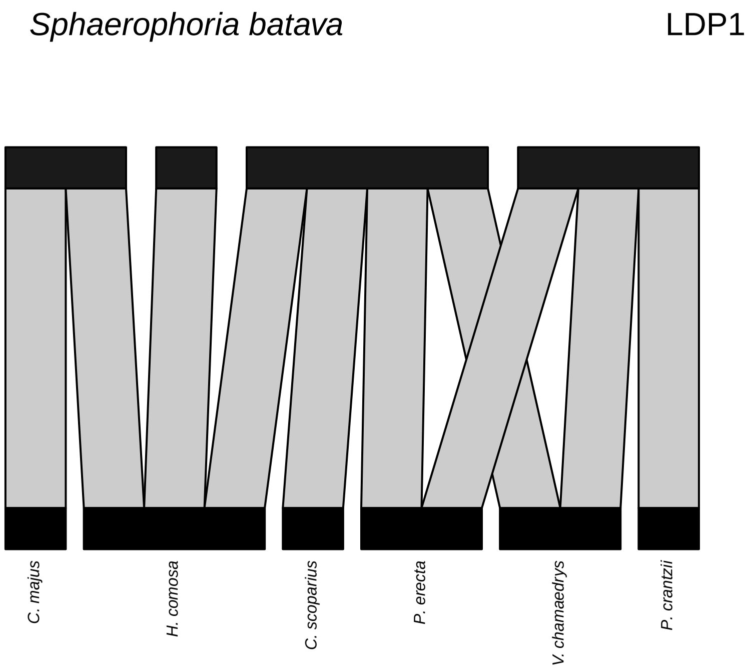
Sphaerophoria batava
LDP1
C. majus
H. comosa
C. scoparius
P. erecta
V. chamaedrys
P. crantzii

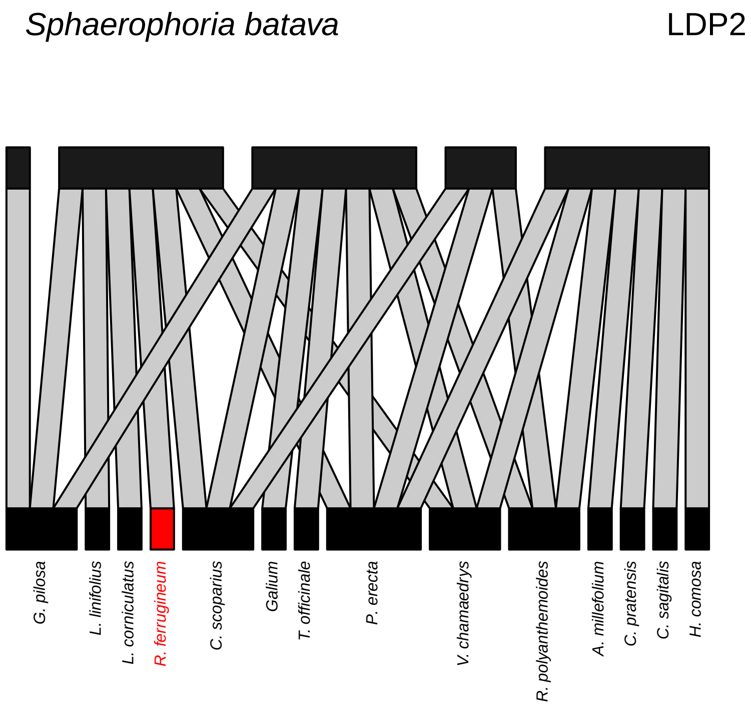
Sphaerophoria batava
LDP2
G. pilosa
L. linifolius
L. corniculatus
R. ferrugineum
C. scoparius
Galium
T. officinale
P. erecta
V. chamaedrys
R. polyanthemoides
A. millefolium
C. pratensis
C. sagitalis
H. comosa

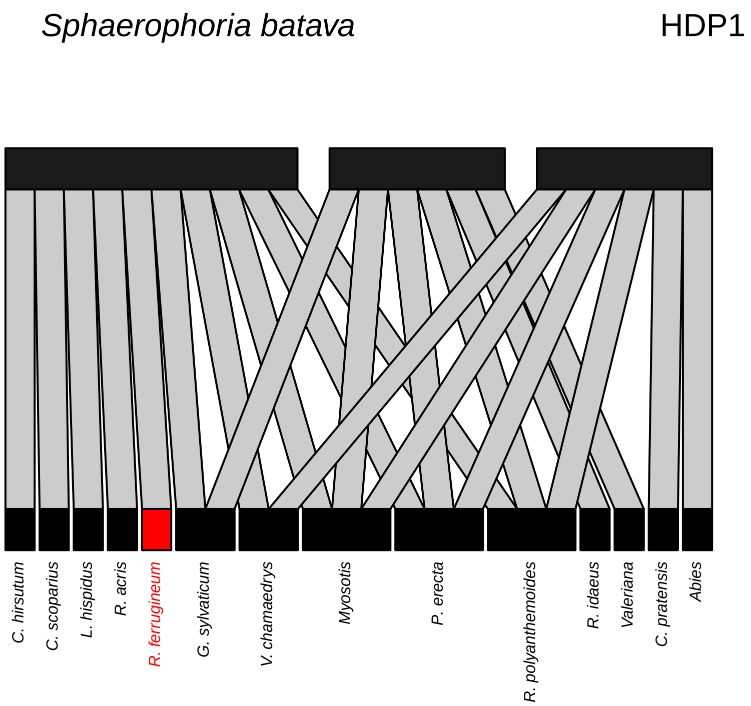
Sphaerophoria batava
HDP1
C. hirsutum
C. scoparius
L. hispidus
R. acris
R. ferrugineum
G. sylvaticum
V. chamaedrys
Myosotis
P. erecta
R. polyanthemoides
R. idaeus
Valeriana
C. pratensis
Abies

Sphaerophoria scripta HDP1

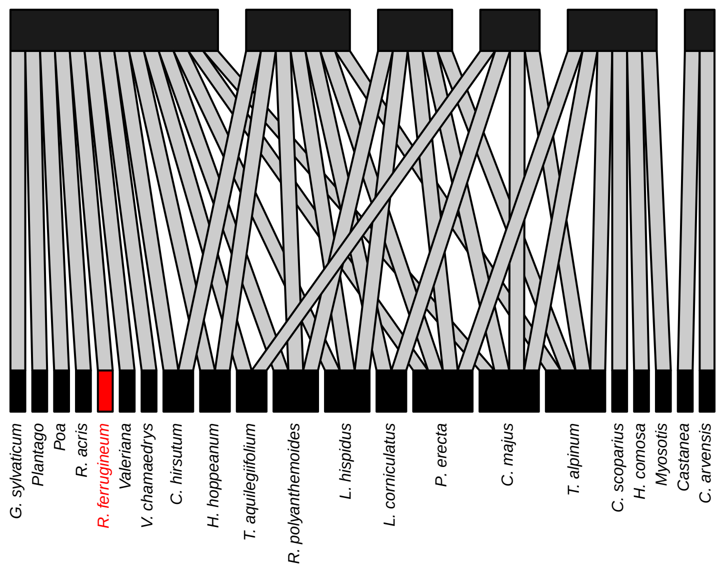

Empis euempis tessellata HDP2

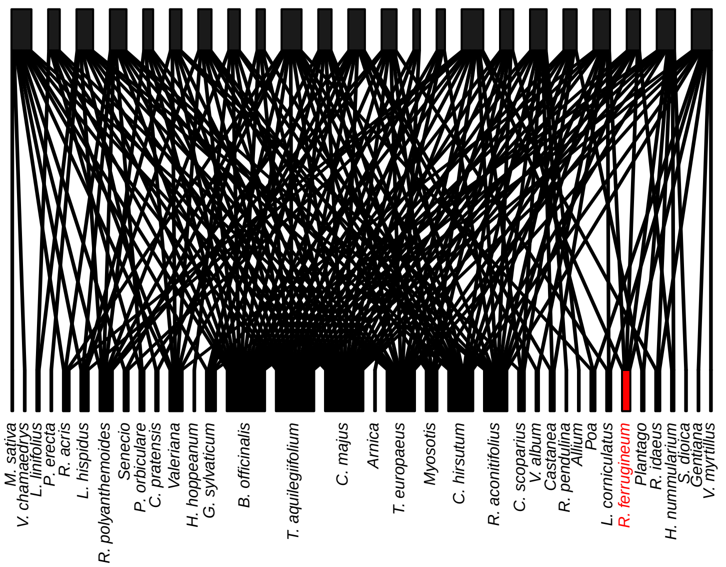

Sphaerophoria interrupta LDP1

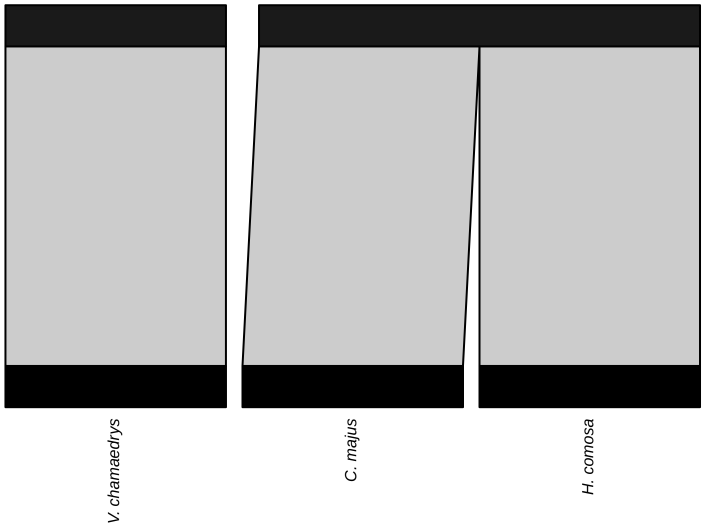

Empis euempis ciliata LDP2

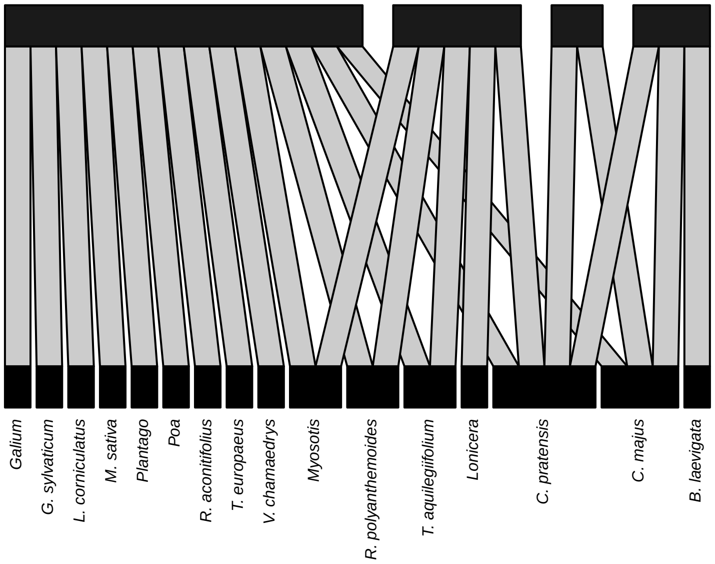

Melanostoma mellinum LDP2

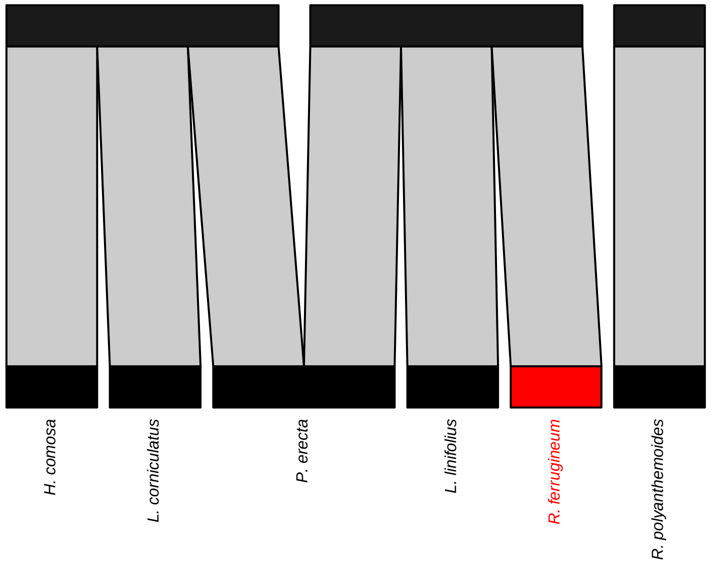

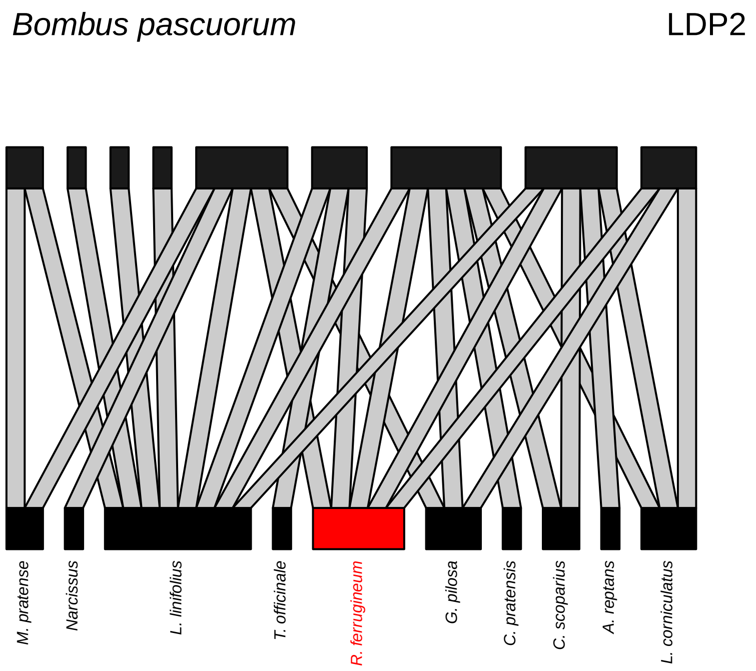

Bombus pascuorum
LDP2
M. pratense
Narcissus
L. linifolius
T. officinale
R. ferrugineum
G. pilosa
C. pratensis
C. scoparius
A. reptans
L. corniculatus

Bombus pratorum LDP2

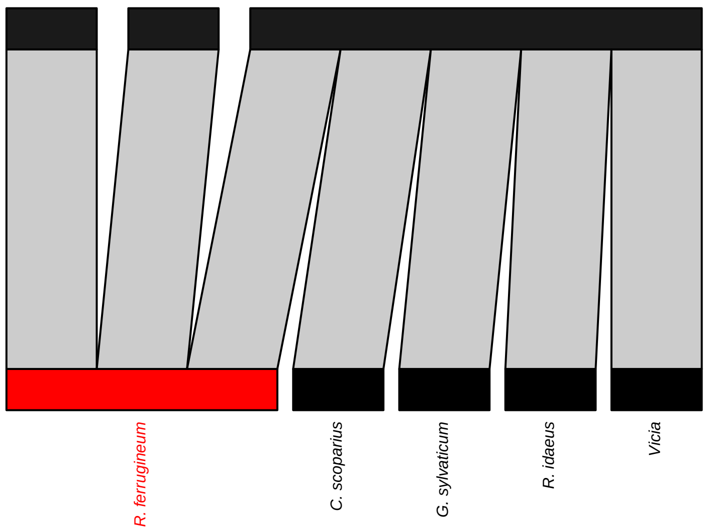

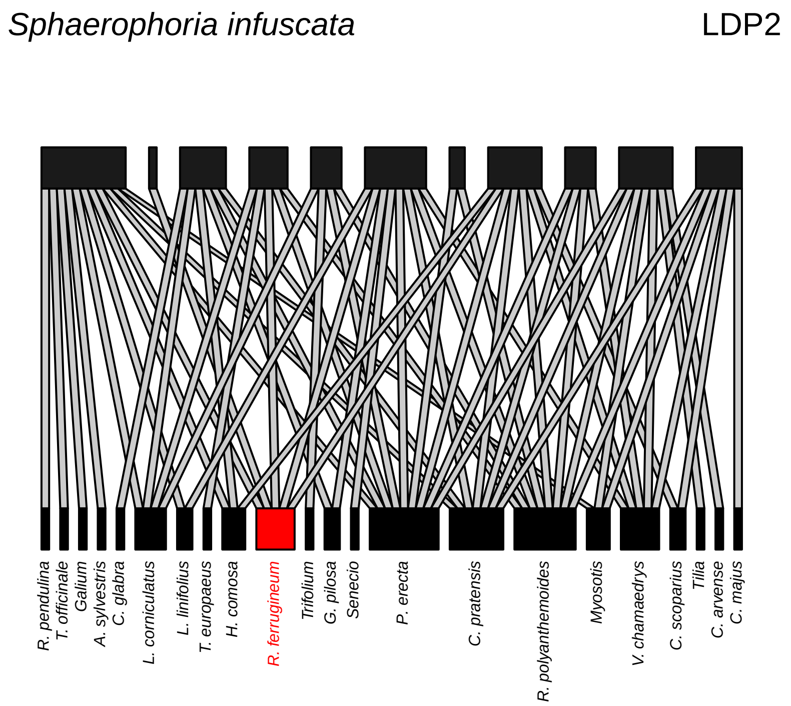
Sphaerophoria infuscata
LDP2
R. pendulina
T. officinale
Galium
A. sylvestris
C. glabra
L. corniculatus
L. linifolius
T. europaeus
H. comosa
R. ferrugineum
Trifolium
G. pilosa
Senecio
P. erecta
C. pratensis
R. polyanthemoides
Myosotis
V. chamaedrys
C. scoparius
Tilia
C. arvense
C. majus
