# Supplementary material for: Pollinator specialization increases with a decrease in a mass‐flowering plant in networks inferred from DNA metabarcoding
Source: Ecol Evol. 2019 Sep 30;9(24):13650–62. doi: 10.1002/ece3.5531 (PMC6953672; doi:10.1002/ece3.5531)
Supplement: Supplementary file 3 [file ECE3-9-13650-s003.docx]

| **Table S1** List of plant and insect species in pollination networks | | | | | | | | |
| --- | --- | --- | --- | --- | --- | --- | --- | --- |
|  | **Family** | | **Insect species** | | | **Family** | **Insect species** | |
| **Hymenoptera** | Apidae | | *Apis mellifera* | | | Andrenidae | | *Andrena sp* |
|  | Apidae | | *Bombus lucorum* | | | Halictidae | | *Halictus sp* |
|  | Apidae | | *Bombus pascuorum* | | | Halictidae | | *Lasioglossum albipes* |
|  | Apidae | | *Bombus pratorum* | | | Halictidae | | *Lasioglossum calceatum* |
|  | Apidae | | *Bombus wurflenii* | | | Halictidae | | *Lasioglossum fratellum* |
|  | Apidae | | *Bombus gerstaeckeri* | | | Unidentified | | *Unidentified* |
|  | Apidae | | *Bombus lapidarius* | | | Tenthredinidae | | *Tenthredo mesomela* |
|  | Apidae | | *Bombus ruderarius* | | | Tenthredinidae | | *unidentified* |
|  | Apidae | | *Bombus soroeensis* | | |  | |  |
|  | Apidae | | *Bombus sylvestris* | | |  | |  |
|  | Apidae | | *unidentified* | | |  | |  |
| **Diptera** | Empididae | | *Empis empis sp* | | | Syrphidae | | *Cheilosia albitarsis ranunculi* |
|  | Empididae | | *Empis euempis ciliata* | | | Syrphidae | | *Cheilosia nigripes* |
|  | Empididae | | *Empis euempis tessellata* | | | Syrphidae | | *Eristalis jugorum* |
|  | Empididae | | *Empis leptempis pandellei* | | | Syrphidae | | *Eristalis nemorum* |
|  | Empididae | | *Empis sp* | | | Syrphidae | | *Eumerus sp* |
|  | Empididae | | *Empis xanthempis testacea* | | | Syrphidae | | *Lapposyrphus lapponicus* |
|  | Empididae | | *unidentified* | | | Syrphidae | | *Melanostoma dubium* |
|  | Syrphidae | | *Sphaerophoria batava* | | | Syrphidae | | *Melanostoma melaria* |
|  | Syrphidae | | *Sphaerophoria infuscata* | | | Syrphidae | | *Melanostoma mellinum* |
|  | Syrphidae | | *Sphaerophoria interrupta* | | | Syrphidae | | *Parasyrphus vittiger* |
|  | Syrphidae | | *Sphaerophoria scripta* | | | Syrphidae | | *Platycheirus albimanus* |
|  | Syrphidae | | *Syrphus ribesii* | | | Syrphidae | | *Platycheirus melanopsis* |
|  | Syrphidae | | *Volucella bombylans* | | | Syrphidae | | *Platycheirus tarsalis* |
|  | Syrphidae | | *unidentified* | | | Syrphidae | | *Rhingia campestris* |
|  | Muscidae | | *Helina reversio* | | | Anthomyiidae | | *Chiastocheta rotundiventris* |
|  | Muscidae | | *Thricops nigritellus* | | | Anthomyiidae | | *Hylemya vagans* |
|  | Calliphoridae | | *Bellardia pandia* | | | Anthomyiidae | | *Pegoplata aestiva* |
|  | Bombyliidae | | *Bombylius major* | | | Agromyzidae | | *Melanagromyza sp* |
|  | Sarcophagidae | | *Sarcophaga subvicina* | | | Sciomyzidae | | *Pherbellia cinerella* |
| **Coleoptera** | Cantharidae | | *Cantharis rustica* | | | Elateridae | | *Ctenicera cuprea* |
|  | Chrysomelidae | | *Cryptocephalus aureolus* | | | Melyridae | | *Dasytes niger* |
|  | Chrysomelidae | | *Cryptocephalus sericeus* | | | Oedemeridae | | *Oedemera virescens* |
| **Lepidoptera** | Nymphalidae | | *Aglais urticae* | | | Lycaenidae | | *Callophrys rubi* |
|  | Nymphalidae | | *Clossiana euprhosyne* | | | Lycaenidae | | *Cupido minimus* |
|  | Nymphalidae | | *Erebia oeme* | | | Pieridae | | *Pieris napi* |
|  | Nymphalidae | | *Coenonympha pamphilus* | | | Hesperiidae | | *Erynnis tages* |
|  | Nymphalidae | | *Euphydryas aurinia* | | | Noctuidae | | *Euclydia glyphica* |
|  | Nymphalidae | | *Melitaea diamina* | | | Geometridae | | *Isturgia sp* |
|  | Nymphalidae | | *Melitaea sp* | | | Geometridae | | *Epirrhoe tristata* |
|  |  | |  | | | L sp. | | *unidentified* |
|  | | **Plant species** | | |  | | |  |
| *Abies* | | *Crepis mollis* | | *Lotus corniculatus* | | | | *Rosa eglanteria* |
| *Agrostis gigantea x A. stolonifera"* | | *Cruciata glabra* | | *Luzula* | | | | *Rosa pendulina* |
| *Achillea millefolium* | | *Cytisus scoparius* | | *Medicago sativa* | | | | *Rubus idaeus* |
| *Ajuga reptans* | | *Dactylis glomerata* | | *Melampyrum pratense* | | | | *Rumex acetosa* |
| *Alchemilla vulgaris* | | *Epilobium montanum* | | *Myosotis* | | | | *Sanguisorba minor* |
| *Allium* | | *Fagus* | | *Narcissus* | | | | *Scrophularia* |
| *Angelica sylvestris* | | *Filipendula ulmaria* | | *Orchidaceae* | | | | *Senecio* |
| *Anthoxanthum* | | *Galium* | | *Pedicularis foliosa* | | | | *Silene dioica* |
| *Anthyllis vulneraria* | | *Genista pilosa* | | *Phyteuma orbiculare* | | | | *Stellaria* |
| *Arnica* | | *Gentiana* | | *Plantago* | | | | *Taraxacum officinale* |
| *Biscutella laevigata* | | *Geranium sylvaticum* | | *Poa* | | | | *Thalictrum aquilegiifoliu* |
| *Bistorta officinalis* | | *Geum rivale* | | *Potentilla crantzii* | | | | *Thesium alpinum* |
| *Capsella rubella* | | *Helianthemum nummularium* | | *Potentilla erecta* | | | | *Thymus serpyllum* |
| *Cardamine pratensis* | | *Hieracium hoppeanum* | | *Prunus* | | | | *Tilia* |
| *Castanea* | | *Hieracium laevigatum* | | *Quercus* | | | | *Tr Trollius europaeus ifolium* |
| *Cerastium arvense* | | *Hippocrepis comosa* | | *Ranunculus aconitifolius* | | | | *Vaccinium myrtillus* |
| *Chaerophyllum hirsutum* | | *Lathyrus linifolius* | | *Ranunculus acris* | | | | *Valeriana* |
| *Chamaespartium sagitalis* | | *Lathyrus pratensis* | | *Ranunculus polyanthemoides* | | | | *Veratrum album* |
| *Conopodium majus* | | *Leontodon hispidus* | | *Rhinanthus alectorolophus* | | | | *Veronica chamaedrys* |
| *Convolvulus arvensis* | | *Lonicera* | | *Rhododendron ferrugineum* | | | | *Vicia* |
