# Supplementary material for: Pollinator specialization increases with a decrease in a mass‐flowering plant in networks inferred from DNA metabarcoding
Source: Ecol Evol. 2019 Sep 30;9(24):13650–62. doi: 10.1002/ece3.5531 (PMC6953672; doi:10.1002/ece3.5531)
Supplement: Supplementary file 4 [file ECE3-9-13650-s004.docx]

| Table S2. Characteristics of species (*sp-sp* networks) and individual (*i-sp* networks) networks based either on the occurrence of plant-insect links (N_link_; left of the slash) or on the sequence counts (N_seq_, right of the slash). Patches with low (LDP1, LDP2) and high (lHDP1, HDP2) floral density. *H*’*_2_*: specialization of network; *d’*: standardized Kullback-Leibler distance measuring the specialization of species; *E*: interaction evenness. * indicate that the networks differed significantly from their corresponding null models (P < 0.05) highlighting higher (+) or lower (-) index values. ns : not significant. | | | | | | | | | | | |
| --- | --- | --- | --- | --- | --- | --- | --- | --- | --- | --- | --- |
|  | No. species or ind. | | Connectance | Mean linkage degree | | | Nestedness | Level of specialization | | | |
|  | Insects | Plants |  | All species | Insects | Plants |  | *H^'^_2_* | *d'* insects | *d'* plants | *E* |
| ***sp-sp* networks** |  |  |  |  | L*_sp_* | L*_p_* |  |  |  |  |  |
| LDP1 | 29 | 39 | 0.12ns/0.12*- | 1.9ns/1.9*- | 4.55ns/4.55*- | 3.3ns/3.4*- | 0.89ns/0.89*- | 0.21*+/0.71*+ | 0.30ns/0.49*+ | 0.31ns/0.57*+ | 0.67*-/0.42*- |
| LDP2 | 25 | 54 | 0.16*-/0.16*- | 2.8*-/2.8*- | 8.8*-/8.8*- | 4.05*-/4.05*- | 0.89ns/0.89*- | 0.19*+/0.51*+ | 0.23ns/0.42*+ | 0.22*+/0.36*+ | 0.70*-/0.49*- |
| HDP1 | 25 | 61 | 0.17*-/0.17*- | 3.1*-/3.1*- | 10.4*-/10.4*- | 4.2*-/4.2*- | 0.83*-/0.83*- | 0.23*+/0.65*+ | 0.28ns/0.53*+ | 0.22*+/0.44*+ | 0.72*-/0.45*- |
| HDP2 | 29 | 61 | 0.16ns/0.16*- | 3.2ns/3.2*- | 10ns/10*- | 4.75ns/4.75*- | 0.88ns/0.88*- | 0.17*+/0.60*+ | 0.21ns/0.49*+ | 0.18*+/0.35*+ | 0.69*-/0.49*- |
| ***i-sp* networks** |  |  |  |  | L*_i_* | L*_p_* |  |  |  |  |  |
| *A. mellifera* |  |  |  |  |  |  |  |  |  |  |  |
| LDP1 | 12 | 4 | 0.31ns/0.31*- | 0.94ns/0.94*- | 1.25ns/1.25*- | 3.75ns/3.75*- | 0.73ns/0.73 | -- /0.61*+ | 0.08*-/0.01*+ | 0*-/0.30*+ | 0.70ns/0.62*- |
| HDP1 | 11 | 23 | 0.2ns/0.2*- | 1.6ns/1.6*- | 4.5ns/4.5*- | 2.2ns/2.2*- | 0.86*+/85.7 | -- /0.87*+ | 0.21*-/0.39*+ | 0.11*-/0.50*+ | 0.71ns/0.39*- |
| HDP2 | 9 | 25 | 0.32ns/0.32*- | 2.1ns/2.1*- | 8ns/8*- | 2.9ns/2.9*- | 0.70*+0.70 | -- /0.62*+ | 0.19ns/0.32*+ | 0.09ns/0.38*+ | 0.79ns/0.36*- |
| *B. lucorum* |  |  |  |  |  |  |  |  |  |  |  |
| LDP1 | 3 | 13 | 0.44ns/0.44*- | 1.1ns/1.1*- | 5.7ns/5.7*- | 1.3ns/1.3*- | 0.79ns/0.79 | -- /0.73*+ | 0ns/0.65*+ | 0.03ns/0.19*+ | 0.77ns/0.55*- |
| LDP2 | 9 | 20 | 0.26ns/0.26*- | 1.6ns/1.6*- | 5.1ns/5.1*- | 2.3ns/2.3*- | 0.80*+/0.80*- | -- /0.68*+ | 0.21ns/0.35*+ | 0.12*-/0.35*+ | 0.74ns/0.45*- |
| HDP1 | 10 | 25 | 0.2ns/0.2*- | 1.5ns/1.5*- | 5.1ns/5.1*- | 2ns/2*- | 0.75ns/0.75 | -- /0.72*+ | 0.29ns/0.49*+ | 0.15ns/0.48*+ | 0.71ns/0.44*- |
| HDP2 | 10 | 26 | 0.23ns/0.23*- | 1.6ns/1.6*- | 5.9ns/5.9*- | 2.3ns/2.3*- | 0.84*+/0.84*- | -- /0.79*+ | 0.22*-/0.66*+ | 0.16ns/0.49*+ | 0.73ns/0.50*- |
| *B. wurflenii* |  |  |  |  |  |  |  |  |  |  |  |
| LDP1 | 5 | 8 | 0.37ns/0.37*- | 1.15ns/1.15*- | 3ns/3*- | 1.9ns/1.9*- | 0.90*+/0.90 | --/0.82*+ | 0.02*-/0.54*+ | 0.04*-/0.38*+ | 0.73ns0.47*- |
| LDP2 | 19 | 17 | 0.20ns/0.20*- | 1.8ns/1.8*- | 3.5ns/3.5*- | 3.9ns/3.9*- | 0.90*+/0.90*- | --/0.73*+ | 0.12*-/0.36*+ | 0.12*-/0.52*+ | 0.73ns/0.44*- |
| HDP1 | 9 | 13 | 0.37ns/0.37*- | 1.9ns/1.9*- | 4.8ns/4.8*- | 3.3ns/3.3*- | 0.71ns/0.70 | --/0.55*+ | 0.17ns/0.28*+ | 0.12ns/0.36*+ | 0.79ns/0.51*- |
| HDP2 | 2 | 4 | 0.87ns/0.87*- | 1.2ns/1.2*- | 3.5ns/3.5*- | 1.75ns/1.75*- | 100ns/100 | --/0.79*+ | 0ns/0.47*+ | 0ns/0.32*+ | 0.93ns/0.52*- |
| *B. pascuorum* |  |  |  |  |  |  |  |  |  |  |  |
| LDP2 | 9 | 10 | 0.3ns/0.3*- | 1.4ns/1.4*- | 3ns/3*- | 2.7ns/2.7*- | 0.83*+/0.83- | --/0.51*+ | 0.12*-/0.24*+ | 0.13ns/0.37*+ | 0.73ns/0.58*- |
| *B. pratorum* |  |  |  |  |  |  |  |  |  |  |  |
| LDP2 | 3 | 5 | 0.47ns/0.47*- | 0.87ns/0.87*- | 2.3ns/2.3*- | 1.4ns/1.4*- | 0.94ns/0.94 | --/0.02*+ | 0ns/0.015*+ | 0.024ns/0.007*+ | 0.72ns/0.19*- |
| *E l. pandellei* |  |  |  |  |  |  |  |  |  |  |  |
| LDP1 | 7 | 12 | 0.24ns/0.24*- | 1.05ns/1.05*- | 2.9ns/2.9*- | 1.7ns/1.7*- | 0.56ns/0.57 | --/0.75*+ | 0.38ns/0.70*+ | 0.17ns/0.56*+ | 0.68ns/0.53*- |
| LDP2 | 15 | 29 | 0.23ns/0.23*- | 2.3ns/2.3*- | 6.7ns/6.7*- | 3.4ns/3.4*- | 0.89*+/0.89*- | --/0.60*+ | 0.17*-/0.37*+ | 0.10*-/0.38*+ | 0.76ns/0.50*- |
| HDP1 | 7 | 21 | 0.33ns/0.33*- | 1.75ns/1.75*- | 7ns/7*- | 2.3ns/2.3*- | 0.69ns/0.69 | --/0.57*+ | 0.19ns/0.35*+ | 0.05*-/0.33*+ | 0.78ns/0.50*- |
| HDP2 | 12 | 32 | 0.24ns/0.24*- | 2.1ns/2.1*- | 7.6ns/7.6*- | 2.8ns/2.8*- | 0.76*+/0.76 | --/0.65*+ | 0.21*-/0.40*+ | 0.11ns/0.44*+ | 0.76ns/0.47*- |
| E. e. ciliata |  |  |  |  |  |  |  |  |  |  |  |
| LDP2 | 4 | 16 | 0.37ns/0.37*- | 1.2ns/1.2*- | 6ns/6*- | 1.5ns/1.5*- | 0.78ns/0.78 | --/0.29*+ | 0.11ns/0.18*+ | 0.08ns/0.11*+ | 0.76ns/0.43*- |
| E. e. tessellata |  |  |  |  |  |  |  |  |  |  |  |
| HDP2 | 23 | 35 | 0.25ns/0.25*- | 3.4ns/3.4*- | 8.7ns/8.7*- | 5.7ns/5.7*- | 0.75*+/0.75 | --/0.33*+ | 0.13*-/0.18*+ | 0.08*-/0.37*+ | 0.79ns/0.60*- |
| *S. batava* |  |  |  |  |  |  |  |  |  |  |  |
| LDP1 | 4 | 6 | 0.42ns/0.42*- | 1ns/1*- | 2.5ns/2.5*- | 1.7ns/1.7*- | 0.75ns/0.75 | --/0.79*+ | 0.21ns/0.72*+ | 0.18ns/0.54*+ | 0.72ns/0.67*- |
| LDP2 | 5 | 14 | 0.36ns/0.36*- | 1.3ns/1.3*- | 5ns/5*- | 1.8ns/1.8*- | 0.75ns/0.75 | --/0.77*+ | 0.15ns/0.61*+ | 0.04*-/0.36*+ | 0.76ns/0.40*- |
| HDP1 | 3 | 14 | 0.52ns/0.52*- | 1.3ns/1.3*- | 7.3ns/7.3*- | 1.6ns/1.6*- | 0.71ns/0.71 | --/0.82*+ | 0.1ns/0.80*+ | 0.06ns/0.30*+ | 0.83ns/0.21*- |
| *S. interrupta* |  |  |  |  |  |  |  |  |  |  |  |
| LDP1 | 2 | 3 | 0.5ns/0.5*- | 0.6ns/0.6*- | 1.5ns/1.5*- | 1ns/1*- | 0.55ns/0.55 | --/1*+ | --/1*+ | 0.33ns/0.5*+ | 0.61ns/0.55*- |
| *S. infuscata* |  |  |  |  |  |  |  |  |  |  |  |
| LDP2 | 11 | 22 | 0.25ns/0.25*- | 1.8ns/1.8*- | 5.5ns/5.5*- | 2.8ns/2.8*- | 0.8*+/0.8*- | --/0.52*+ | 0.20ns/0.42*+ | 0.13ns/0.42*+ | 0.75ns/0.56*- |
| *S. scripta* |  |  |  |  |  |  |  |  |  |  |  |
| HDP1 | 6 | 21 | 0.3ns/0.3* | 1.4ns/1.4*- | 6.3ns/6.3*- | 1.8ns/1.8*- | 0.63ns/0.63 | --/0.80*+ | 0.32ns/0.75*+ | 0.15ns/0.44*+ | 0.75ns/0.53*- |
| *V. bombylans* |  |  |  |  |  |  |  |  |  |  |  |
| LDP2 | 2 | 8 | 0.5ns/0.5*- | 0.8ns/0.8*- | 4ns/4*- | 1ns/1*- | 0.36ns/0.36 | --/1*+ | --/1*+ | 0.12ns/0.30*+ | 0.75ns/0.23*- |
| HDP1 | 3 | 13 | 0.38ns/0.38*- | 0.94ns/0.94*- | 5ns/5*- | 1.15ns/1.15*- | 0.57ns/0.57 | --/0.73*+ | 0.33ns/0.75*+ | 0.15ns/0.38*+ | 0.74ns/0.51*- |
| HDP2 | 13 | 32 | 0.25ns/0.25*- | 2.3ns/2.3*- | 8.1ns/8.1*- | 3.3ns/3.3*- | 0.78*+/0.78*- | --/0.63*+ | 0.18*-/0.45*+ | 0.12ns/0.41*+ | 0.77ns/0.48*- |
| *M. mellinum* |  |  |  |  |  |  |  |  |  |  |  |
| LDP2 | 3 | 6 | 0.39ns/0.39*- | 0.78ns/0.78*- | 2.3ns/2.3*- | 1.2ns/1.2*- | 0.55ns/0.61 | --/0.68*+ | 0.33ns/0.75*+ | 0.17ns/0.44*+ | 0.67ns/0.46*- |
