# Supplementary material for: Pollinator specialization increases with a decrease in a mass‐flowering plant in networks inferred from DNA metabarcoding
Source: Ecol Evol. 2019 Sep 30;9(24):13650–62. doi: 10.1002/ece3.5531 (PMC6953672; doi:10.1002/ece3.5531)
Supplement: Supplementary file 5 [file ECE3-9-13650-s005.pdf]

Appendix S1 Indices for quantifying individual specialization and niche components.

For each patch, we completed plant species-pollinator individual matrices (M = $[n_{ij}]_{IxP}$; P: plants, I: insects) with n_{ij} being either the larger number of either *trnL* P6 loop or *ITS1* sequences (quantitative M_{seq}) or the presence/absence (1; 0 otherwise) of sequences (qualitative M_{link}) of the plant species *j* yielded from the pollen load of the individual insect *i*. From those matrices we calculated:

(1) niche components, i.e. the total niche width (TNW), the variation in pollen type use within individuals (within-individual component WIC), the variation in pollen type use among individuals (between-individual component, BIC; with TNW= WIC+BIC)

$$WIC = \sum_i p_i \cdot (-\sum_j p_{ij} \cdot \ln p_{ij})$$

$$BIC = -(\sum_i p_i \cdot \ln p_{ij}) - [\sum_j q_j \cdot (-\sum_i \gamma_{ij} \cdot \ln \gamma_{ij})]$$

$$TNW = -\sum_j q_j \cdot \ln q_j$$

$$p_{ij} = n_{ij} / \sum_j n_{ij} \qquad p_i = \sum_j n_{ij} / \sum_i\sum_j n_{ij} \qquad q_j = \sum_i n_{ij} / \sum_i\sum_j n_{ij} \qquad \gamma_{ij} = n_{ij} / \sum_i n_{ij}$$

p_{ij} = proportion of pollen type *j* in the pollen load of individual pollinator *i*.

p_i = proportion of all pollen types used by species x.

q_j = proportion of pollen type *j* in the pollen load of species x

γ_{ij} = proportion of total pollen type *j* used by species x and carried by individual *i*.

(2) The proportional niche similarity between individuals and their populations (PS_i):

$PS_i = 1 - 0.5 \cdot \sum_j | p_{ij} - q_j |$ where p_{ij} and q_j are the same as above.

(3) the inter-individual (IO) food niche overlap :

$$IO = \sum_i\sum_j w_{ij} / a \cdot n \cdot (a-1)$$

Where w_{ij} is the number of pollen types (identified by metabarcoding) shared between two individual pollinators, a the number of conspecific individuals of the pollinator species x, n the number of plant species visited by the pollinator species x.

(4) the species food niche overlap (SPO)

$$SPO = \sum_{j} w_{ij} / p \cdot (s\text{-}1)$$

Where w_{ij} is the same as in IO, p is the total number of pollen types (plant species) in the community and s the total number of insect species.
